# The plant cell wall—dynamic, strong, and adaptable—is a natural shapeshifter

**DOI:** 10.1093/plcell/koad325

**Published:** 2024-02-01

**Authors:** Deborah Delmer, Richard A Dixon, Kenneth Keegstra, Debra Mohnen

**Affiliations:** Section of Plant Biology, University of California Davis, Davis, CA 95616, USA; BioDiscovery Institute and Department of Biological Sciences, University of North Texas, Denton, TX 76203, USA; MSU-DOE Plant Research Laboratory, Michigan State University, East Lansing, MI 48823, USA; Complex Carbohydrate Research Center and Department of Biochemistry and Molecular Biology, University of Georgia, Athens, GA 30602, USA

## Abstract

Mythology is replete with good and evil shapeshifters, who, by definition, display great adaptability and assume many different forms—with several even turning themselves into trees. Cell walls certainly fit this definition as they can undergo subtle or dramatic changes in structure, assume many shapes, and perform many functions. In this review, we cover the evolution of knowledge of the structures, biosynthesis, and functions of the 5 major cell wall polymer types that range from deceptively simple to fiendishly complex. Along the way, we recognize some of the colorful historical figures who shaped cell wall research over the past 100 years. The shapeshifter analogy emerges more clearly as we examine the evolving proposals for how cell walls are constructed to allow growth while remaining strong, the complex signaling involved in maintaining cell wall integrity and defense against disease, and the ways cell walls adapt as they progress from birth, through growth to maturation, and in the end, often function long after cell death. We predict the next century of progress will include deciphering cell type–specific wall polymers; regulation at all levels of polymer production, crosslinks, and architecture; and how walls respond to developmental and environmental signals to drive plant success in diverse environments.

## Teaser

This review provides a historical context for the processes by which plant cell wall structures are created, assemble, sense, and respond to signals and change during progression from birth to death.

## Introduction

In 1665, Robert Hooke peered through his primitive microscope at a slice of cork and described little boxes he called “cellula”—rooms that monks inhabited. These “cellula” were dead cells, and all that remained visible to him were their cell walls. For centuries after, much of what we knew about cell walls was derived from what was visible with our own eyes, either viewed directly or through the ever-improving technique of light microscopy. Form helps predict function, and we now know that cell walls play a key role in determining cell, tissue, and organ shapes. Botanists, amazed by the diverse types of cells found in plants, watched the ways in which cells with thin “primary” cell walls (PCWs) first begin to form a new wall at the time of cell division and how cell and overall plant growth is tied to the expansion of such thin walls. Cell diversity is also intimately linked to the way cells mature, building “secondary” cell walls (SCWs) that thicken to form structures that provide strength to stems to withstand loss of turgor, protect against water loss and diseases, and function in transport of water and minerals from roots to leaves. The overall shape of a plant and its organs, in particular flowers, was found to be one of best predicters of ancestry, and taxonomy became one of the most popular (and controversial) fields for botanists extending well into the early 20th century. Smith (1962) provides an absolutely delightful and colorful history of botany including the hilarious struggles to develop the field of botanical taxonomy.

In this centennial celebration year recognizing the founding of ASPB, we have aimed to provide a historical context to the process of discovery and to emphasize milestones that made a difference in moving the field forward. The first part of the review presents the progression of learning about the structure, biosynthesis, and functions of the 5 major types of wall polymers: cellulose, hemicelluloses, pectins, wall-associated proteins, and lignin. The second part aims to integrate this knowledge of individual polymers and delve deeper into some of the fascinating challenges faced by plant cell walls. We examine models that have attempted to explain the still-challenging problem of how the polymers found in PCWs can be assembled into a structure strong enough to resist high turgor pressure while allowing expansion to permit growth. Recognizing that walls are not passive structures but constantly communicating and adapting to serve the needs of the cells they protect, we then discuss the rapidly expanding field of cell wall signaling. We end with an examination of the life of the cell wall from birth to survival even beyond the death of their parent cells.

Because each of us has focused most of their career on one of the major wall polymers, we consider this a rare opportunity to integrate our collective knowledge gathered over many years. Aware that new ideas do not always pan out, we also take this opportunity to offer a few new ideas of our own and end with our assessment of major challenges for the future of cell wall research.

**Figure 1.**
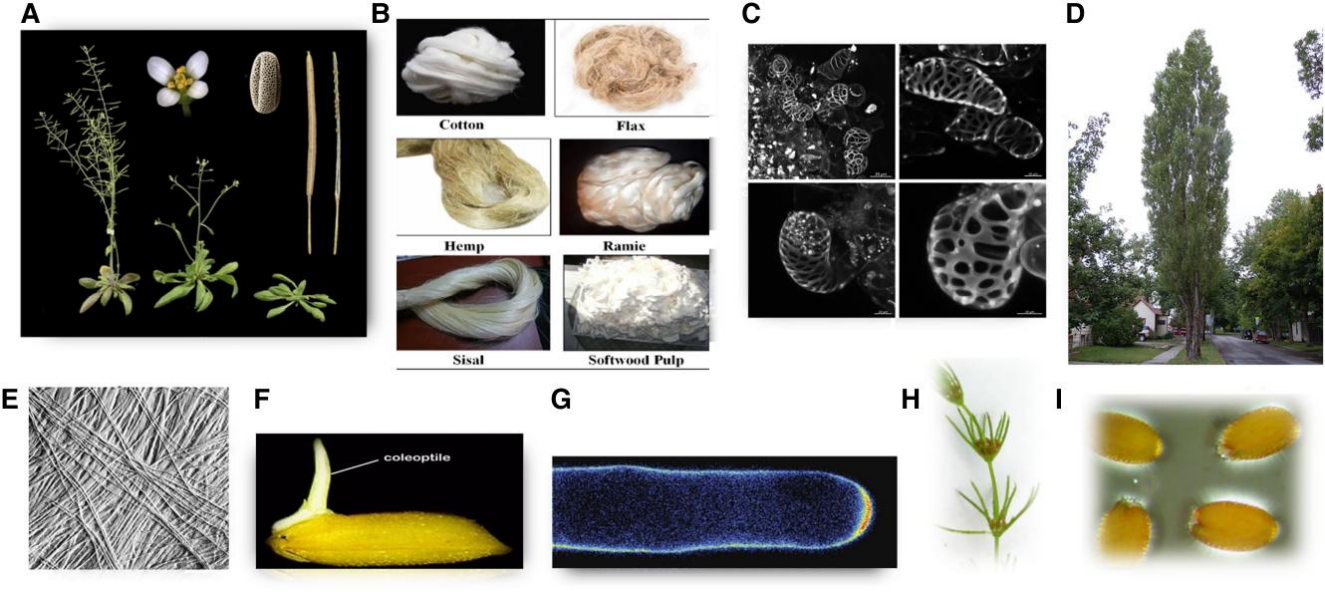
Some model systems that are especially useful for the study of plant cell walls. **A)**  *Arabidopsis thaliana*; its small size, short generation time, small genome, and ease of mutant selection make this a favorite for genetic studies (Liepman et al. 2010). **B)** Fiber cells that have SCWs that are cellulose-rich and nearly free—or with much reduced levels—of hemicelluloses and lignin include cotton fibers (actually epidermal hairs), bast fibers (especially from flax and ramie), and fibers of tension wood and are favorites for studying cellulose. Ramie (*Boehemeria nivea*) cellulose is favored by x-ray crystallographers for its clear diffraction patterns; cotton fibers are single cells that show synchronous development within a single boll, and their mature SCWs have >90% cellulose (Haigler et al. 2012). **C)** Suspension-cultured cells from the C4 plant sugarcane (*Saccharum* hybrid spp; example shown taken from Simões et al., 2020) or C3 plants such as Zinnia elegans (reviewed by Demura, 2013) can be induced to differentiate into tracheary elements, making them good models for study of xylem biogenesis. **D)** Hybrids of poplar (*Populus*) have become models of choice for trees due to ease of propagation, molecular breeding, transformation, and large genome resources (Tuskan et al. 2004). **E)** Onion epidermal cells (shown here are surface cellulose microfibrils [MFs]) can be easily isolated as a living, hormone-responsive monolayer, especially useful for studying PCW expansion (Suslov et al. 2009; Zhang et al. 2021c). **F)** A popular model for grass walls of very different Type II PCW structure is the maize coleoptile (Carpita 1984). **G)** Pollen tubes of Lily have been used to elucidate the oscillating nature of tip growth (McKenna et al. 2009) and discovery of key signaling cascades (Li et al. 2016a). **H)** Charophycean algae have played a key role in studying the role of pectins in diffusive cell wall elongation (Proseus and Boyer 2012b). **I)** Some mucilage-secreting cells (MSCs) such as those from Arabidopsis, are rich in pectins, whereas others are rich in only one kind of hemicellulose such as xyloglucan, xylan, or glucomannan, making MSCs valuable for studies on biosynthesis and wall polymer interactions (Arsovski et al. 2010). All figures are licensed through the Creative Commons and used with permision.

Not all cells that build walls do so the same way, so we must emphasize the importance of using a variety of model systems (Fig. 1), *Arabidopsis thaliana*, certainly, but our discussion just might include some of the botanists’ favorite very odd-shaped cells.

## The 5 major polymers of the plant cell wall

### Cellulose

Common claims that cellulose “is the most abundant organic compound on earth” and that taking density differences into consideration, it is “stronger than steel” are actually true (see McNamara et al. 2015). Yet cellulose, the cell wall polymer with the simplest basic structure—one type of sugar joined by 1 type of linkage—is anything but simple given the variety of structures that can be assembled from individual β-1,4-glucan chains. We will see that this feature allows it to interact, not only with itself but with other noncellulosic polysaccharides, and its presence is so important for wall structure that a decrease in cellulose level in the wall sends out distress signals that alert the cell to quickly do whatever possible to correct this situation. Beyond its role in the plant, all these properties make cellulose a key component of timber, textiles, paper, and a major feedstock for chemicals; and the fact that it is difficult to digest influences agriculture and global nutrition.

#### Structure of cellulose

##### The early studies

Early botanists often equated the wall with the term cellulose because of its abundance in many cell types. Anselme Payen (1838), “the father of cellulose chemistry,” isolated the major sugar of cell walls and was surprised to find that its elemental composition was the same as that of starch. Payen recognized that cellulose could be broken down to glucose but had no idea of its structure nor how the monomers were aggregated to create a polymer so very different from starch. Decades later, the sequential efforts of some towering figures in the emerging fields of sugar and polymer chemistry led to the conclusion that cellulose is a polymer of thousands of glucose residues linked in β-1–4 configuration (Fischer 1902; Haworth et al. 1927, 1931; Staudinger 1932). The concept of a polymer was novel to chemistry at the time, as the length of 1 type of polymer could vary considerably even as the chemical formula stayed the same; but by 1936, the idea became generally accepted (Kanaya and Kaji 2016). Estimates of chain lengths ranged from 100s to many thousands of residues depending on the source and how the cellulose was isolated (Delmer 1983).

The Swiss scientist Albert Frey-Wyssling defined a new field he termed “submicroscopic morphology” that led to several insightful predictions concerning the highly oriented and crystalline properties of cellulose (Frey-Wyssling and Ambronn 1927; Frey-Wyssling 1939). Using the newly introduced electron microscope, Frey-Wyssling's predictions were confirmed, and he and Fritz Muhlethaler provided our best early images of cellulose fibrils in native cell walls. One major conclusion was that fibrils of varying widths consisted of smaller fibrils of about 35 Å width, corresponding roughly to a 6×6 array of chains (Mühlethaler 1960; Frey-Wyssling and Mühlethaler 1963). This so-called “elementary fibril” (or microfibril [MF]) is now considered a fundamental unit of cellulose, although the predicted number of chains has been reduced over time. The Federal Institute of Technology Zurich under Frey-Wyssling also pioneered the technique of freeze-fracture (Moor et al. 1961) that led much later to the first images of cellulose synthase complexes (CSCs).

It took more than 60 years to answer the key question of whether the chains of native cellulose (cellulose I) were parallel (reducing ends all in the same direction) or anti-parallel (Kroon-Batenburg and Kroon 1997; French 2000; Zugenmaier 2021). Better computer modeling improved analyses of diffraction patterns, and, working independently with the same sample of the alga Valonia native cellulose I, Gardner and Blackwell (1974) and Sarko and Muggli (1974)—and later Woodcock and Sarko (1980) with fibers of ramie (*Boehmeria nivea*)—all concluded that the chains were indeed parallel. This was confirmed by other methods (Hieta et al. 1984; Chanzy and Henrissat 1983), and the parallel chain arrangement for cellulose I is now the accepted model. Cellulose II, produced by alkali treatment of native cellulose, is a more stable form, and the early findings of Meyer and Misch (1937) that the chains are in anti-parallel arrangement were confirmed later by Kolpak and Blackwell (1976) and Buleon and Chanzy (1978).

##### New technologies provide new insights

**Figure 2.**
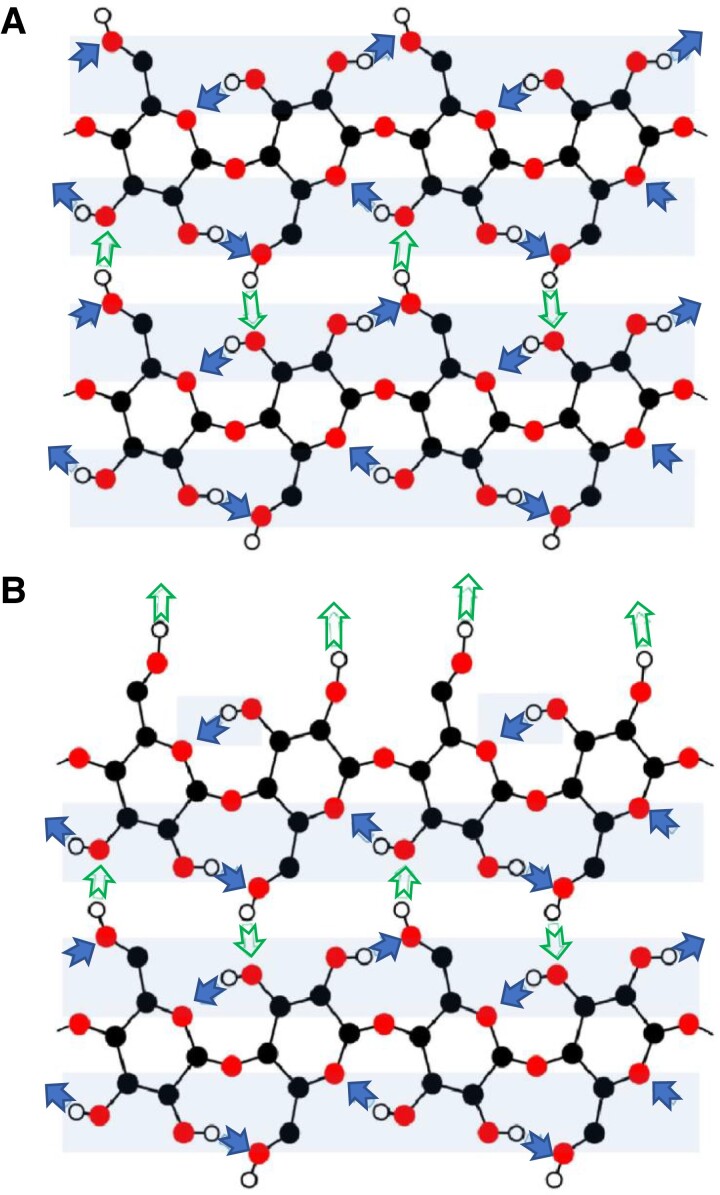
Hydrogen bonding schemes for cellulose. **A)** A 2-chain segment of “crystalline” cellulose with all C-6 in the *tg* conformation, permitting a line of intramolecular hydrogen bonds (shaded arrows) that run along each side of each cellulose chain (lightly shaded horizontal bands). Intermolecular hydrogen bonds are shown as open arrows. **B)** Two-chain segment of “surface” cellulose with the edge of the upper chain having C-6 in the *gt* conformation, so that the line of intramolecular hydrogen bonding is interrupted and there is an increased number of transversely oriented, intermolecular hydrogen bond sites (top row of open arrows) that allows for interaction with water, other MFs or other polymers. Modified from Jarvis (2018) by permission of the author.

It took crystallographers so long to agree on the crystal structure of native cellulose largely because native cellulose can exist in many crystalline forms that show only subtle differences in x-ray diffraction. There has since been a dramatic rise in the number of new technologies that can be applied to study cellulose structure (Jarvis 2013, 2018). According to Jarvis (2018), “Broadly, NMR spectroscopy is most informative about chain conformation; crystallography, about chain packing; Fourier transform infrared spectroscopy, about hydrogen bonding; atomic force microscopy (AFM), about microfibril dimensions; small-angle scattering, about the aggregation of MFs.” Using cross-polarization magic angle NMR, Atalla and Vanderhart (1984) demonstrated that native cellulose can exist in at least 2 crystalline forms: Iα, found in native bacterial and algal cellulose, and Iβ, found in tunicates and higher plants. Different conformations at C-6 in the crystalline core (Fig. 2A) versus surface MFs (Fig. 2B) can create altered patterns for hydrogen bonding that alter the potential for surface chains to interact with other MFs or other polymers.

The widely cited work of Nishiyama et al. (2002) showed 2 parallel chains in pure Iβ having slightly different conformations, and neutron scattering supported 2 different types of intrachain hydrogen bonding between 1 glucose residue with each of its neighbors that stabilizes a co-planar orientation of glucose. Strikingly, no hint of inter-sheet O—H…O bonds was found, indicating that sheets of cellulose must be held together primarily by van der Waals forces along with some weak C—H…O bonds (see also Notley et al. 2004; McNamara et al. 2015). Thus, the glucan chains have a hydrophilic and a hydrophobic face that allow for very strong interchain interaction, although each individual interaction is weak.

Surprisingly, the cellulose of maize (*Zea mays*) PCWs consists of up to 7 different allomorphs, only 1 of which is almost identical to either Iα or Iβ (Wang et al. 2016b). Some allomorphs show differences on the surface of the MF and were suggested to be possible targets for interaction with other polymers such as pectins. Others show interior chains associated with surface chains, whereas another is embedded in the core of the MF. Yet another is predicted to be on the surface but poorly hydrated and was suggested as a possible target for expansins (see below). How the surfaces of MFs interact with water, with themselves to create larger bundles or with other wall polymers, is critical to overall wall structure and mode of expansion (see below) and is still an ongoing field of investigation (Cosgrove 2022; Jarvis 2023).

#### Biosynthesis of cellulose

It is hard to document all the progress that is being made in this field, so it is fortunate that many details have been well-reviewed by others (Wallace and Somerville 2014; McNamara et al. 2015; Turner and Kumar 2018; Wilson et al. 2021). In particular, we leave detailed discussion of CESA (cellulose synthase) trafficking, some aspects of regulation, and the evolution of the genes/proteins involved to others (Haigler and Roberts 2019; Lampugnani et al. 2019; Polko and Kieber 2019). Hopefully our historical perspective provides examples that might inform future work.

##### The early years

Luis Leloir, an Argentine biochemist, physician, and pioneer of carbohydrate biosynthesis, made several truly milestone discoveries. Key was the discovery in the 1950s of nucleoside diphosphate sugars and recognition that the free energy of hydrolysis releasing the sugar was sufficient for them to serve as donors of sugars for polysaccharide synthesis. Discovery of UDP-Glc in 1950 was only part of many that led to the Nobel Prize (see Leloir 1983). UDP-Glc is a key substrate in polysaccharide biosynthesis, either directly for cellulose, callose (β-1,3-glucan), and hemicellulosic glucans or indirectly as precursor to other NDP-sugars that are themselves substrates for synthesis of noncellulosic polysaccharides (Bar-Peled and O’Neill 2011).

The obligate aerobic bacterium *Acetobacter xylinum*, lacking flagellae, secretes cellulose in order to float and gain oxygen in liquid environments (Hestrin 1962). Glaser (1958) showed that it uses UDP-Glc as substrate for cellulose synthesis in vitro, albeit at quite low rates. Moshe Benziman's group later achieved high rates in vitro through the important discovery of cyclic-di-GMP as a potent activator (Aloni et al. 1982; Ross et al. 1987). One gene (*BcsA*) in a 4-gene operon encodes the catalytic subunit (Saxena et al. 1990); the other genes (Saxena et al. 1994; Römling and Galperin 2015) encode proteins that assist in binding of c-di-GMP to BcsA (*BcsB*), create a pore for MF extrusion through the outer membrane (*BcsC*), or have endoglucanase activity (*BcsD*) that, along with other proteins (Abidi et al. 2022), are important for determining crystallinity of the product.

Another prominent biochemist, Zev Hassid, believed in 1957 that it would only be a short time before his laboratory showed how cellulose was synthesized in plants. He set 2 of his students to work using mung beans expecting to find that UDP-Glc was substrate. The students returned with news that the product was not cellulose but callose (β-1-3-glucan). Hassid could not believe it, and the poor students had to repeat their results several more times before it was published (Feingold et al. 1958; the students, Liz Neufeld and David Feingold, became very well-known polysaccharide biochemists in spite of abandoning cellulose synthesis as a target!). This frustration was repeated by many other laboratories, finding that the bulk of the product in vitro was all or mostly callose (Robinson and Quader 1981; Delmer 1983). Callose synthesis in vitro generally requires 2 activators; micromolar levels of Ca^2+^ and any 1 of a variety of β-glucosides (Hayashi et al. 1987), and assays for cellulose synthase often inadvertently contained both because cellobiose was often supplied and micromolar Ca^2+^ is present in plant extracts if not specifically chelated. Vincent Bulone stands out as one who often reported successful in vitro synthesis of cellulose and deserves much credit for providing protocols for assays of glycosyltransferases (GTs) in plants (Brown et al. 2012). Callose synthases are very stable enzymes in contrast to the quite labile plant CSCs. More recently, Oehme et al. (2019) and Purushotham et al. (2022) showed that only minor changes in amino acid sequences and conformation of glycosyltransferase-2 (GT2) enzymes as classified by the Carbohydrate-Active Enzyme Database (CAZy) (Cantarel et al. 2009; Drula et al. 2022) can lead to β-1,3, as opposed to β-1,4-linkages. One wonders why plants, having evolved CSLF and CSLH enzymes (also GT2 proteins, see Hemicellulose section) that can make glucan backbones with both linkages, have never evolved a callose synthase analogous to the GT2 bacterial β-1,3-glucan (curdlan) synthase (Oehme et al. 2019). Instead, plant callose synthases evolved from yeast/fungi and are quite different in structure (Schneider et al. 2016).

##### Early contributions from microscopy

Preston (1964)—another force in the world of cell wall research—speculated that a plant CSC is a complex of catalytic subunits in which the number of subunits dictates the number of chains produced in a single MF. Malcolm Brown—a genius at capturing images of the process of cellulose synthesis—showed pictures of *A. xylinum* extruding cellulose from a linear array of pores (Brown et al. 1976). Other freeze-fracture images supported Preston's vision, showing huge multi-subunit “terminal complexes” (TCs) at the ends of very large growing MFs in algae like Oocystis and Valonia (Montezinos and Brown 1976; Itoh and Brown 1984; Brown 1996). Surprisingly, some of these TCs were aligned next to each other and were clearly moving in opposite directions, creating an arrangement that could lead to more stable antiparallel interactions between the MFs; this bi-directional mobility was also later seen for plant CSCs (Paredez et al. 2006). Arabidopsis mutants defective in phosphorylation of the complex lose this property, which affects MF bundling and stem phenotype (Chen et al. 2016). This raises the question of whether anti-parallel regions of MF bundling might be the “hot-spots” for expansin function proposed by Cosgrove (2022) (see later). Jarvis (2023) comments that it is not known to what extent MFs bundle in either the parallel or more stable antiparallel orientations, but the issue would certainly seem to deserve further study (Makarem et al. 2020). For plants, a more modest complex was observed in the plasma membrane of maize (Mueller and Brown 1980) and tracheary elements of cress (Herth 1985) showing 6-fold symmetry and was termed a rosette (probably by Andrew Staehelin according to Nick Carpita). These generally appear as solitary structures in plants, and Giddings et al. (1980) demonstrated remarkable arrays of up to 175 rosettes in the plasma membrane of the alga Micrasterias.

##### CesA genes in plants

The first plant *CESA* gene was not identified until 1996 (Pear et al. 1996). In cotton (*Gossypium hirsutum*) fibers, the rate of cellulose synthesis increases more than 100-fold during the transition from PCW to SCW (Meinert and Delmer 1977), and random cDNA sequencing of a transition stage cDNA library found 2 very similar cDNAs containing scattered sequences characteristic of processive GT2 enzymes and other regions showing strong sequence similarity to the transmembrane helices (TMHs) of the BcsA protein (Pear et al. 1996). But the cotton genes also possessed an N-terminal domain (NTD) encoding a ring-finger domain, a highly plant-conserved region (PCR) and another region of variable sequence (HVR, later called CSR for class-specific region). The latter 2 insertions split the conserved sequences required for catalytic activity, thus explaining why the genes had not been discovered earlier. The cotton genes were strongly induced at the onset of SCW synthesis (Pear et al. 1996), but no genetic evidence was presented to confirm that they encoded cellulose synthases.

Chris Somerville and Herman Hofte had the vision to identify Arabidopsis (Fig. 1) as a model plant in which specific mutations in cellulose synthesis could be easily selected. Williamson's laboratory in Australia isolated a temperature-sensitive mutant of Arabidopsis termed *rsw1* impaired in the synthesis of PCW cellulose (Arioli et al. 1998). It quickly became evident that the *RSW1* locus had a very close relationship to that of the cotton fiber genes and encoded what we now refer to as *AtCESA1* that functions in synthesis of PCW cellulose. And so…off to the races! Richmond and Somerville (2000) showed that Arabidopsis has 10 *CESA* genes. Also identified were a number of related cellulose-synthase-like (*CSL*) families, some of which function in hemicellulose synthesis (see below). The *CSLD* family members are closest to the *CESAs* (Doblin et al. 2002), and reports increasingly suggest that at least some CSLD proteins serve an alternate function to the CESAs for synthesis of cellulose (see below). CSLD3 was recently verified to possess β-1,4-glucan synthase activity and is most similar in structure to CESA6 (Yang et al. 2020b).

A complete CSC in vivo involves more than just CESA proteins (Lampugnani et al. 2019). An endoglucanase, KORRIGAN, analogous to BcsD, may play a role in cleaving disordered chains during synthesis (Nicol et al. 1998; Vain et al. 2014), although its role remains enigmatic because a mutation in the predicted catalytic site still complemented the *kor* mutant phenotype (Lei et al. 2014). Also unclear is the exact function of COBRA, a glycosylphosphatidylinositol (GPI)-anchored protein that is apparently associated with the CSC and affects crystallinity (Schindelman et al. 2001; Roudier et al. 2005). A better understanding of the functions of KOR and COBRA is needed. Furthermore, many scientists seem to have forgotten that freeze-fracture studies of rosettes showed protein globules on the opposite face of the PM that matched in position the central core of the rosettes on the opposite membrane face (Giddings et al. 1980; Mueller and Brown 1980), suggesting the central globule as 1 possible location for accessory proteins. CESAs may also be regulated through phosphorylation and S-acylation that may affect activity, rosette structure, and/or recycling (Kumar et al. 2016; Polko and Kieber 2019).

##### Comparisons of bacterial and plant CESA structures

Study of *CESA* mutants of Arabidopsis has led to 2 surprising but important conclusions: the rosette responsible for synthesis of the fundamental MF is comprised of 2 classes defined largely, but not exclusively, by the homology of their CSRs; and each class contains 3 necessary but non-identical subunits, usually (but not always) in 1:1:1 ratio. Class I proteins (CESA1, CESA3, and any one of CESA 2, 5, 6, or 9) are used primarily for PCW synthesis, whereas Class 2 (CESA 4, 7, and 8) are necessary for SCW synthesis (Turner and Kumar 2018). For clarity, the names of all plant homologs of the Arabidopsis CESAs in other plants have been changed in this review to match those used for Arabidopsis.

A significant milestone came when Jochen Zimmer's group crystallized a catalytically active bacterial BcsA-BcsB protein complex and determined its structure to 3.25-A resolution (Morgan et al. 2013; McNamara et al. 2015); a cello-oligosaccharide remained in the structure during crystallization. In this proposed structure, BcsA contains 4 TMHs in the amino-terminal region and 4 in the carboxy-terminal region, separated by a cytoplasmic intracellular loop that forms the catalytic domain that is protected by a cyclic-di-GMP–regulated gating loop that controls access to the active site (Morgan et al. 2014). The channel formed by TMHs 3–8 accommodates the translocating glucan. The overall structure resembles other membrane-integrated, processive GT2s such as hyaluronic acid synthase and chitin synthase (Bi et al. 2015).

**Figure 3.**
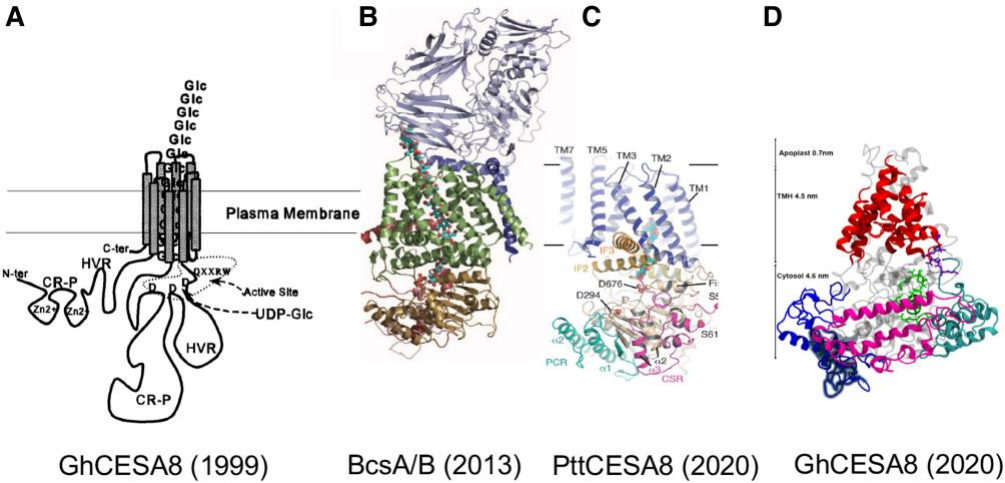
Comparison of models of cellulose synthase catalytic subunits. **A)** Cartoon model taken from Delmer (1999) shows how the active site in plants is recreated to resemble that of BcsA by looping out of CSR (HVR) and PCR (CR-P) domains. **B)** BcsA/B structure determined by X-ray diffraction from its crystal structure (Morgan et al. 2013). **C** and **D)** PttCESA8 modeled from cryo-electron microscopy of reconstituted recombinant protein (Purushotham et al. 2020) and cotton fiber GhCESA8 (original name GhCESA-1) computationally modeled from the sequence (Singh et al. 2020). All models possess an elongated shape with a central catalytic domain (brown in BcsA; wheat in PttCESA8; grey and green in GhCESA) and a channel comprised of TMHs (green, blue, red, moving L to R) through which the growing glucan chain is extruded through the inner/plasma membrane. The PCR (aqua in PttCESA8; magenta in GhCESA8), CSR domains (magenta in poplar; aqua in cotton), and NTD (shown in blue only in GhCESA8) are plant specific and not found in BcsA. The BcsB domain associated with BcsA (blue gray) is not found in plants.

Significant advances have been made in producing catalytically active recombinant CESAs; PpCESA5 from moss (*Physcomitrella patens*) and PttCESA8 from hybrid poplar (*Populus tremula* × *tremuloides*) synthesized very thin β-1,4-glucan fibrils in vitro in the absence of added primer (Purushotham et al. 2016; Cho et al. 2017). When isolated from insect cells, PttCESA8 reconstituted into structures that largely ranged from monomer to trimer, and a homotrimer fraction was size-selected and analyzed by cryo-electron microscopy (Purushotham et al. 2020). In Fig. 3, a model of the proposed monomer of PttCESA8 is compared with that of other proposed catalytic subunit structures, including BcsA, and, for historical purposes, the original crude model (Delmer 1999) that was based on simple domain structure analyses.

Although differing in many details, all models show that the TMHs form transmembrane channels, and the catalytic domain of plant CESAs is reconstructed by looping out into the cytoplasm of the PCR and CSR domains that are proposed to function as sites for CESA-CESA interactions and/or necessary accessory proteins. Possible modes of regulation of the plant gating loop are under study (Olek et al. 2023; Verma et al. 2023). Binding of c-di-GMP regulates catalytic activity through the gating loop in bacteria but not plants (Morgan et al. 2014), and this loop is predicted to collapse into the region of the active site when the CSC is inactive in order to maintain PM membrane potential. Older work by Bacic and Delmer (1981) and Delmer et al. (1982) indicated that glucan synthesis in membrane vesicles of cotton or intact *A. xylinum* is inhibited by collapsing the delta psi (electric component), but not delta, pH component of the membrane potential and perhaps offers some clues in this regard.

Initiation of synthesis without added primer (see above) may put to rest the suggestion that sitosterol glucoside can serve as the primer for cellulose synthesis in cotton fibers (Peng et al. 2002). Cotton fibers clearly make sterol-di-tri- and tetra-cello-oligosaccharides, but attempts to demonstrate their initiator activity in Arabidopsis have failed. However, it does appear that CSCs function in sterol-rich portions of the plasma membrane (Schrick et al. 2012; Turner and Kumar 2018), another promising area for further study.

##### MF structure predicts rosette structure (or vice versa)?

Current thinking considers 2 different models for the way in which the 3 different CESAs required for synthesis of PCW or SCW assemble into rosettes. One model proposes that each of 2 of the 6 lobes are comprised of CESA homotrimers that alternate positions within the rosette; alternatively, each of the 6 lobes is assembled into identical heterotrimers, wherein each CESA in the lobe has a unique role. As of this writing, there is no clear answer, but homotrimers may be more likely because recombinant SCW CESA8 (and more recently CESA7; Zhang et al. 2021b) can so easily form active homotrimers, although no complete rosette has yet been assembled. Confusingly, recombinant rice (*Oryza sativa*) CESA8 CatD domain can show clear redox-dependent dimerization (Olek et al. 2014), whereas Vandavasi et al. (2016) obtained trimers from a similar recombinant AtCESA1. Analysis of the PCR crystal structure (Rushton et al. 2017; Zhang et al. 2021b) fitted well with the trimeric structure of PttCESA8s proposed by Purushotham et al. (2020). However, studies of the CSR domain by Scavuzzo-Duggan et al. (2018) and Olek et al. (2023) suggest dimerization through key cysteine residues. Kurek et al. (2002) showed redox-dependent formation of either homo- or heterodimers of the ring fingers of cotton fiber CESAs, and the NTD domain is clearly essential for activity, although it has not proven possible to model trimers with dimerized ring fingers.

The answers may lie in the way trimers are assembled into rosettes. Wilson et al. (2021) further examined the idea that redox-dependent CSR dimerization is one stabilizing link between trimers. Examining their models for a dimer of trimers, one can see several possibilities where ring-finger dimers could also form and act as stabilizing trimer-trimer associations, a possibility that deserves further consideration. Dimers clearly predominate in decomposing CSCs from some cells such as cotton fibers (Kurek et al. 2002; Wen et al. 2022), and it was reported that when GhCESA7 was knocked out, the CSC degraded to GhCESA4–8 dimers, while knocking out GhCESA8 led to GhCESA4–7 dimers (Wen et al. 2022). CESAs 4, 7, and 8 can dimerize with each other in a redox-dependent manner, and this interaction was proposed to occur before assembly into a complete rosette (Altanassov et al. 2009). Whether one gets dimers or trimers in decomposing rosettes may depend on the relative stability of the redox-dependent linkages found in trimers vs possible dimerizations that link trimers into rosettes.

The current conclusion is that the basic PCW MF is usually comprised of 18 chains (Jarvis 2013; Newman et al. 2013; Nixon et al. 2016; Ye et al. 2018; Haigler and Roberts 2019; Song et al. 2020). Models suggest assembly into MFs might be based on 5 layers of chains in a 34,443 arrangement (Kubicki et al. 2018) or 6 layers of 234,432 chains (Song et al. 2020). However, a 24-chain model has been convincingly proposed for spruce (Fernandes et al. 2011), and recent work strongly supports this model for other wood SCW MFs as well (Tai et al. 2023). Haigler and Roberts (2019) have written an excellent analysis of the evolution of rosettes and TCs and the relationship between structure of the MFs and the rosettes.

##### The role of cortical microtubules in orientation of MFs

Perhaps the most exciting contribution from microscopy that opened a whole new approach to studying cellulose synthesis is the early work of Paredez et al. (2006). A DNA construct encoding AtCESA6 was fused to a fluorescent protein and was active when transformed into a mutant *cesA6* background, and mobile CSC complexes were visualized in hypocotyl epidermal cells. They moved at a constant speed corresponding to an addition of 300 to 1,000 Glc residues per minute and displayed a short residence time in the PM of 7 to 10 min—roughly the amount of time to synthesize 1 PCW glucan chain. Movement was often bidirectional and paralleled the orientation of aligned cortical microtubules beneath the PM. One can only imagine how the early pioneers of cytoskeletal biology would have reacted to actually watching the process of MT-aligned MF synthesis in real time. Although the residence time of PCW CesAs is short, other work indicates that the CesA proteins are quite stable, suggesting they may be recycled (Hill et al. 2018). In contrast, SCW CesAs of cotton fibers showed half-lives of less than 30 minutes (Jacob-Wilk et al. 2006).

The residence time of a CSC was calculated to exceed that of the underlying dynamic MTs, and it was observed often that the MT would disappear, but the CSC would continue its forward motion in the same direction, confirming that CSC movement is not assisted by some MT-motor protein mechanism but rather by the force of polymerization of the MFs. Later, Chan and Coen (2020) studied CSC movement after MT disappearance and confirmed that CSCs continued in the same direction for the residence time of the CSC, after which new CSCs appeared that followed the same trails as previous ones; this pattern continued apparently guided by the orientation of the nascent MFs in the wall. This kind of behavior has been seen in other situations in vivo; for example, the pattern of cellulose deposition during xylem vessel development is initially determined by MT orientation but persists long after MT disruption (Roberts et al. 2004; Schneider et al. 2017; Watanabe et al. 2018). Such observations have occasionally been used as an argument that MTs may not be so important for directing CSC motion. However, the MT-directed path takes preference because, when a CSC encounters an MT, the CSC preferentially begins following the new MT orientation. These studies clearly support the notion that the primary mechanism for orientation of MFs, a major event in the anisotropic elongation of plant cells, is controlled by the orientation of cortical MTs. But what controls reorientation of the MTs? One intriguing finding is that the localized de-methylesterification of pectins in the wall predicts and precedes the pattern adopted by the MTs (Peaucelle et al. 2015). Other evidence indicates that actin microfilaments interact with MTs and may play a special role in targeting of CSC and MF orientation (especially in SCW synthesis) (Lei et al. 2012). MT orientation may occur by sensing the direction of tensile stress (Hamant et al. 2019), and recent work indicates that the tethering of CSCs to MTs disturbs this stress-induced MT reorganization (Schneider et al. 2022).

The approach conceived in 2006 to follow rosette movement in vivo has also revealed important information on CSC trafficking (Lampugnani et al. 2019). CSCs assemble in the Golgi and are transported to the PM, where they become tethered to cortical MTs through binding of the CESA NTD to POM-POM2/Cellulose Synthase Interacting proteins (Gu et al. 2010; Bringmann et al. 2012). Once activated, the CSCs stall after 7 to 10 min and then are endocytosed. CESAs then transiently accumulate in small CESA compartments or are degraded in the lytic vacuole. While active, the forward movement of the CSC might be expected to displace the MTs from their PM location, but this has been shown to be prevented by cortical MT uncoupling proteins that regulate this interaction (Liu et al. 2016). Other older data may still be relevant: the cellulose synthesis inhibitor 2,6-dichlorobenzonitrile (DCB) somehow may act through disruption of MT organization, and an 18- to 20-kD MT-associated protein (MAP20) binds an active photo-affinity analog of DCB (Delmer et al 1987; Rajangam et al. 2008), consistent with a complex interaction between synthesis of cellulose and orientation of MTs (Schneider et al. 2017). However, genetic evidence that MAP20 is the target of DCB action in vivo is still lacking. Also fascinating is the finding that although kinesins do not control CSC movement, they are involved in movement on MTs of noncellulosic polysaccharide cargos from the Golgi to the PM, and 1 specific kinesin, referred to as Fragile Fiber1, has recently been shown to interact with cortical MT uncoupling proteins. This interaction is proposed to facilitate the localized deposition of matrix polymers in proximity to the sites where cellulose is deposited, representing a potentially important way to coordinate the deposition of cellulose with that of noncellulosic polymers (Ganguly et al. 2020).

### Hemicelluloses

#### Structures of hemicelluloses

**Figure 4.**
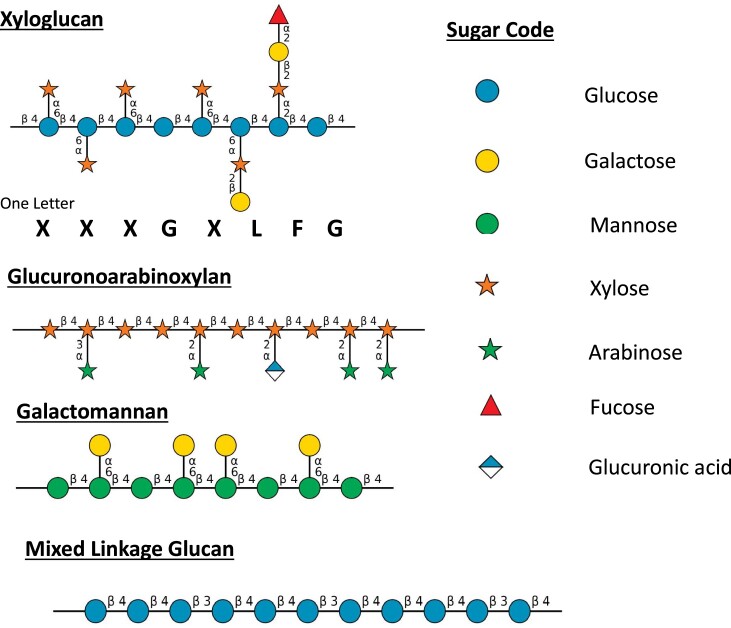
Representative structures of hemicelluloses. The xyloglucan fragment is typical of dicot PCWs. The galactomannan is typical of those found in legume seeds. The glucuronoxyloarabinan is typical of those found in grass PCWs. The structures were drawn using software described by Cheng et al. (2017).

Hemicellulosic polysaccharides consist of a diverse collection of different polymers including xyloglucans (XyG), xylans, mannans, and mixed-linkage glucans (MLGs) (Fig. 4). Providing a precise definition of a hemicellulose is difficult. The common feature is that they bind to cellulose. Most have a backbone with β-1,4-linked sugars, but as the name implies, MLG also has β-1,3-linkages in the backbone. The backbones of most hemicelluloses have additional sugars and/or acetyl groups attached, but again MLG is the exception (Fig. 4).

When considering matrix polysaccharides, both hemicelluloses and pectins, it is important to remember that the structures depicted in figures are a representation of many different molecules. Polysaccharides are different from nucleic acids and proteins, where the sequence of monomers is determined by the genetic code. In contrast, the structure of polysaccharides is determined by the specificity of the enzymes that synthesize them, and not all individual molecules in a collection of polysaccharides have the same structure. Another difference is that sugars have multiple hydroxyl residues where a neighboring sugar, or an acetyl residue, can be attached. Thus, determining the structure of a polysaccharide requires complex chemical and analytical methods.

##### Xyloglucans

Among hemicelluloses, XyGs have the most complex structures (Fig. 4). Like cellulose, they have a backbone of β-1,4–linked glucosyl residues but with α-linked xylosyl residues attached in a regular pattern to the 6-position of most glucosyl residues (Fig. 4). Many xylose residues are further substituted, most frequently with galactosyl or galactosyl plus fucose residues. A shorthand notation for the substitution pattern of each location along the chain uses a single letter designation (Fig. 4) (Fry et al. 1993). Other substitution patterns are found in XyGs from various plant species, with the patterns sometimes varying among different tissues within a species (Hayashi 1989; Scheller and Ulvskov 2010; Schultink et al. 2014; Pauly and Keegstra 2016). XyG is the most abundant hemicellulose in the PCWs of gymnosperms, dicots, and noncommelinid monocots. Plants with XyG as the most abundant hemicellulose are said to have Type I primary walls (Carpita and Gibeaut 1993).

##### Xylans

Xylans have a pentose-based backbone of β-1,4–linked xylosyl residues linked in a way that allows binding to cellulose. The backbone is substituted with α-linked arabinose residues, α-linked glucuronic acid resides, and variable numbers of acetyl esters (Fig. 4). Xylans, as opposed to XyGs, are the most abundant hemicellulose in the PCWs of grasses; these walls are categorized as Type II (Carpita and Gibeaut 1993). The distribution of the side-chain residues varies depending on the plant species, the plant tissue, and the developmental stage of the tissue (Scheller and Ulvskov 2010). In addition, xylans from gymnosperms and selected angiosperms have a unique tetrasaccharide located at the reducing end of the polysaccharide, although the function of this sequence of 4 sugars is unknown (Smith et al. 2017). The distribution and patterns of substitution along the xylan backbone has functional significance. For example, the backbone substitution patterns of SCW xylans impact their interactions with cellulose (Busse-Wicher et al. 2016; Grantham et al. 2017). More recently, Tryfona et al. (2023) demonstrated that grass cell walls have xylans with at least 3 different substitution patterns and suggest that these patterns influence the way in which xylans interact with other wall components. For example, an arabinoxylan with evenly distributed arabinofuranosyl residues and lacking glucuronic acid facilitates binding to cellulose while other substitution patterns may facilitate interactions with other wall components such as lignin (Tryfona et al. 2023). Xylans are the most abundant hemicellulose in the SCWs of all angiosperms. Because of their abundance in SCWs of woody plants, there are massive quantities of xylans in the biosphere; it has been estimated that 10 billion tons of carbon are incorporated into xylans annually (Smith et al. 2017).

##### Mannans

Mannans are taxonomically the most widely distributed and evolutionarily the most ancient of the hemicelluloses (Voiniciuc 2022). They contain either a β-1,4–linked mannan backbone or a backbone consisting of both β-1,4–linked mannose and glucose. Some have been found to have alternating mannose and glucose in the backbone (Yu et al. 2022). The backbone sugars are substituted with α-linked galactose attached to the 6-position of backbone mannose (Fig. 4). Some mannans have very few or no side chains, whereas other galactomannans are heavily substituted with galactose, thereby producing polysaccharides with very different physical properties. For example, the unsubstituted mannans in ivory nut (from *Phytelephas* species) are insoluble and can be used as carving material for artwork. In some algae, unsubstituted mannans can substitute for cellulose (Scheller and Ulvskov 2010). On the other hand, highly substituted galactomannans are water soluble and can produce viscous solutions with food product and industrial applications (Sharma et al. 2022). Mannans are generally very minor components of PCWs and SCWs of most angiosperms but are frequently the most abundant hemicellulose in the SCWs of gymnosperms.

##### Mixed-linked glucans (MLGs)

MLG has a simple structure with no side chain residues (Fig. 4). It is less widely distributed in the plant kingdom than other hemicelluloses, being found mainly in the primary cell walls of the grasses plus in the cell walls of a few lower plants and some fungi (Burton and Fincher 2009). The β-1,3 linkages create kinks in an otherwise straight polymer so that the frequency and spatial distribution of β-1,3 linkages determines the physical properties of the polymers (Burton and Fincher 2009).

#### Hemicelluloses as storage compounds in plant seeds

Grant Reid and his colleagues significantly contributed to our understanding of plants that use hemicelluloses as reserve polysaccharides in their seeds. In his 1985 review, he pointed out that studies began late in the 19th century, when “…it was clearly recognized by botanists that the massively thickened cell walls present in many seeds contained reserve substances” (Reid 1985). Different plant species utilize different polysaccharides as storage reserves; for example, tamarind (*Tamarindus indica*), nasturtium (*Tropaeolum majus*), and many other species store XyG as a reserve polysaccharide. On the other hand, fenugreek (*Trigonella foenum-graecum*), coffee (*Coffea arabica*), and many other species store polymers in the mannan family, especially galactomannans that have received significant attention because of their industrial and medical applications (Sharma et al. 2022). Their unique rheological properties cause them to have wide use in the food, pharmaceutical, and cosmetic industries, as well as in hydraulic fracturing fluid (Sharma et al. 2022). *Dendrobium catenatum*, containing large quantities of glucomannan, has been extensively studied for centuries and utilized as both food and a Chinese herbal medicine (Qi et al. 2022).

MLG is also found as a reserve polysaccharide in the seeds of cereal grains (Morrall and Briggs 1978). Use of MLG as a reserve polysaccharide has been studied in detail in the model grass *Brachypodium distachyon*, where it is present in thick endosperm cell walls and is mobilized during seedling germination (Francin-Allami et al. 2023). Although xylans are also found in seeds of some plants, the arabinoxylan from the seed husks of *Plantago ovata* has received the most attention (Belorio and Gomez 2022). This psyllium mucilage consists of a highly branched β-1,4–linked xylan backbone (Fischer et al. 2004) and assists *P. ovata* seeds with hydration and gemination. It has been widely used as fiber in the human diet.

As methods for investigating polysaccharide structure were developed in the middle of the 20th century, it was recognized that storage polysaccharides, primarily from seeds (Kooiman 1957) but also from other organs, were similar in structure to hemicelluloses from plant cell walls. For example, XyG from seeds (Kooiman 1967), extracellular polysaccharides of suspension-cultured sycamore (*Acer pseudoplatanus*) cells, and PCWs (Becker et al. 1964; Aspinall et al. 1969; Bauer et al. 1973) all had similar structures.

Because seed reserve and mucilage polysaccharides can be isolated more easily and using milder conditions, they have provided valuable insight into the structure of the various hemicelluloses. As described below, these seed systems have been exploited to identify the genes and proteins involved in the biosynthesis of cell wall polysaccharides. Because the presence of wall polysaccharides as reserve polymers has arisen independently several times during evolution, analysis of the seed systems provides an opportunity to understand how polysaccharide synthesis is regulated. For example, the entire pathway from sucrose to galactomannan in fenugreek is upregulated during seed formation (Wang et al. 2012). Such systems should provide opportunities to identify transcription factors controlling this process.

#### Biosynthesis of hemicelluloses

The biosynthesis of matrix polysaccharides, both hemicelluloses and pectins, takes place in the Golgi. It involves a series of complex processes that generally require a different biosynthetic enzyme for every different linkage found in each polymer (Lerouxel et al. 2006). Results over the last 2 decades have revealed that plant cells employ 3 different strategies for the synthesis of matrix polysaccharide backbones. The first is exemplified by MLG that is synthesized by CSLFs, proteins that are closely related to CESA and CSLD proteins (Yin et al. 2014; Little et al. 2018). CSLF is a glycan synthase (Purushotham et al. 2022) that operates similarly to the CESA proteins described earlier. The second strategy is illustrated by mannans and XyG, which are synthesized by CSLA and CSLC, respectively (Dhugga et al. 2004; Cocuron et al. 2007). These proteins are also glycan synthases but are distant relatives of CESA (Yin et al. 2014; Little et al. 2018). They also differ from the CESA, CSLD, and CSLF proteins in that they operate in concert with GTs that add side chains to the growing backbone (Pauly and Keegstra 2016; Zabotina et al. 2021; Voiniciuc 2022). The third strategy is fundamentally different in that the backbone of xylans and pectic polysaccharides are synthesized by complexes of glycosyltransferase that operate in the lumen of the Golgi (Ye and Zhong 2022; Anders et al. 2023). In these cases, complexes of GTs assemble the backbone (Engle et al. 2022; Ye and Zhong 2022; Anders et al. 2023) while different GTs add the side chain residues when present (Smith et al. 2017; Ye and Zhong 2022).

Over the past 2 decades, investigators have exploited the systems that produce large quantities of individual polysaccharides described above to identify the genes and proteins responsible for their synthesis in a manner analogous to the studies with cotton that led to the identification of the CESA proteins (Pear et al. 1996). Dhugga and colleagues (2004) used developing guar (*Cyamopsis tetragonoloba*) seeds that store large quantities of galactomannan to identify the *CSLA* genes responsible for the synthesis of the mannan backbone. Cocuron et al (2007) used developing nasturtium (*Tropaeolum* spp.) seeds that produce large quantities of XyG to identify the *CSLC* genes responsible for XyG backbone synthesis. For the synthesis of the backbone of rhamnogalacturonan I (RG-I) (see section on pectins below), Takenaka et al (2018) investigated mutant Arabidopsis plants that had defects in mucilage production to identify a rhamnosyltransferase required for RG-I synthesis. This strategy has also been used to identify the GTs required for the addition of side chain sugar residues (Edwards et al. 1999; Jensen et al. 2012, 2013).

Because GTs are mostly type II membrane proteins where the active site is in the Golgi lumen, they utilize nucleotide sugars present inside the lumen. Thus, Golgi-localized sugar nucleotide transporters are required during polysaccharide synthesis. As noted earlier, the various NDP sugars are made from UDP-Glc via a complex set of pathways present in the cytoplasm or the Golgi membranes (Bar-Peled and O’Neill 2011). On the other hand, glycan synthases, including most CSL proteins, have multiple membrane spans; these enzymes accept sugars from nucleotide sugars in the cytosol and, during synthesis, transport the nascent polymer to the lumen of the Golgi, where GTs add side chain sugars as needed. The newly synthesized polysaccharides move from the Golgi lumen to the cell surface via membrane vesicles, which fuse with the plasma membrane, thereby releasing the polymers into the wall matrix. However, very little is known about how the Golgi-synthesized polysaccharides are assembled into a functional cell wall. A summary of the biosynthesis of each type of hemicellulose is presented below.

##### Mannans

Soon after the discovery of mannan synthase (Dhugga et al. 2004), Liepman et al. (2005) demonstrated that several members of the Arabidopsis *CSLA* gene family had mannan or glucomannan synthase activity when expressed in cultured insect cells. Subsequently, *CSLA* gene family members from other species were shown to be responsible for mannan biosynthesis, and it has been postulated that all *CSLA* genes encode mannan synthase (Liepman and Cavalier 2012; Voiniciuc 2022). Of note is that GDP-Man and GDP-Glc, as opposed to UDP-Glc, are used for backbone synthesis.

##### Xyloglucan

Expression of cDNA clones from both nasturtium and Arabidopsis *CSLC* genes in *Pichia pastoris* resulted in the cells producing β-1,4–linked glucan (Cocuron et al. 2007), leading to the conclusion that both likely encode the glucan synthase that makes the XyG backbone. Because *Pichia* does not produce UDP-xyl, it was not possible to confirm these glucan synthases were responsible for XyG biosynthesis, although several lines of evidence supported this conclusion. Later, Kim et al. (2020) demonstrated that a quintuple mutant with disruptions in all 5 Arabidopsis *CSLC* genes has undetectable XyG levels, thereby providing compelling evidence that *CSLC* genes are responsible for XyG biosynthesis. The phenotypes of these and other mutants lacking XyG are described below.

The biosynthesis of mannans and XyGs is similar in many ways (Liepman and Cavalier 2012). With respect to backbone synthesis, the *CSLA* and *CSLC* gene families have sequence similarities and a common evolutionary origin while being evolutionarily distinct from other subgroups in the CESA superfamily (Yin et al. 2014; Little et al. 2018). Little et al. (2018) suggest this lack of connection reflects “…the likely dual endosymbiotic origin of the superfamily” with *CSLA/CSLC* coming from 1 event, whereas *CESA*, *CSLD*, and *CSLF* came from a different event. Side chains attached to the 6-position of each polymer are added by enzymes present in CAZy family GT34 that have both sequence and biochemical similarities (Edwards et al. 1999; Faik et al. 2002; Cavalier and Keegstra 2006). Yu et al. (2022) have elaborated on the similarities in the structure and biosynthesis of the 2 polymers, with evidence that they may have similar functions in that mannans may be able to substitute for XyG in mutants lacking XyG.

As noted above, XyG has a complex array of side chain substitutions. Work in the last 2 decades has produced considerable knowledge about the genes and proteins responsible for the addition of these side chains. Because this work is well documented in several recent reviews (Scheller and Ulvskov 2010; Schultink et al. 2014; Pauly and Keegstra 2016; Julian and Zabotina 2022), here we briefly highlight the important role played by genetics, both forward and reverse, in identifying not only the genes and proteins responsible for side chain addition but also the functions of the polysaccharides and their side chains.

The story begins in Chris Somerville's laboratory, where several Arabidopsis mutants with alterations in cell wall composition were identified (Reiter et al. 1997). One of them, *mur2*, a mutant with reduced levels of fucose, led to identification of the gene responsible for the addition of the fucose side chains on XyG (Perrin et al. 1999; Vanzin et al. 2002). A second, the *mur3* mutant, was later shown to encode a galactosyltransferase that adds galactose to XyG at the location where fucose is attached (Fig. 4) (Madson et al. 2003; Kong et al. 2015). Reverse genetics was also used to isolate mutant plants with disruption of the genes encoding XyG xylosyltransfereases (XXT) (Cavalier et al. 2008; Zabotina et al. 2012). As described in more detail in a later section, these mutant lines also aided in evaluating the function of XyG.

##### Mixed-linked glucans (MLGs)

Early studies on the biosynthesis of MLG focused on confirming that it was made by a unique enzyme. As noted above, early studies on cellulose biosynthesis identified enzyme preparations that made both β-1,3 and β-1,4 linkages. The question was whether MLG was made by the combined action of 2 different enzymes or a unique enzyme that made both linkages. Bruce Stone was a memorable contributor to plant cell wall biochemistry. Both funny and feisty, he possessed encyclopedic knowledge of β-glucans in plants and managed to exhaust 2 sets of authors who worked with him writing 2 volumes on the subject. Bruce and his colleagues resolved the question of 1 or 2 enzymes by identifying and partially characterizing an enzyme activity that produced MLG (Smith and Stone 1973; Henry and Stone 1982a, 1982b). Many years later, Stone was coauthor of a manuscript reporting the identification of the *CSLF* genes and proteins responsible for MLG biosynthesis (Burton et al. 2006). Doblin et al. (2009) provided evidence that the grass-specific *CSLH* genes also encode proteins capable of MLG synthesis. Both the *CSLF* and *CSLH* genes are more closely related to *CESA* genes than to *CLSA* and *CSLC* genes (Yin et al. 2014; Little et al. 2018). Although many questions remain about the details of MLG biosynthesis, recent in vitro studies using the barley (*Hordeum vulgare*) CSLF protein have demonstrated that 1 enzyme can synthesize both linkages found in MLG. Molecular modeling and mutagenesis identified a region of the protein near the cytoplasmic side of the glucan channel that controls the frequency of β-1,3 links in the polymer product (Purushotham et al. 2022).

##### Xylan

As noted above, xylan biosynthesis is very different from the biosynthesis of the other hemicelluloses. Most of the genes encoding xylan synthase proteins were identified through forward genetic screens as mutations that altered xylem function, called *irregular xylem* or *irx* mutants. The xylan backbone is synthesized by a complex of GTs and related proteins that either have a single transmembrane domain or that lack a transmembrane domain. The proteins encoded by these genes belong to families GT47 and GT43 in the CAZy classification scheme. Xylan backbone synthesis requires the cooperation of 3 different nonredundant proteins that make up the xylan synthase complex. They include IRX10, or related proteins such as IRX10L, belonging to CAZy family GT47 plus IRX9 and IRX14, or related proteins belonging to CAZy family GT43 (Smith et al. 2017; Ye and Zhong 2022; Anders et al. 2023). The exact role of each of these proteins in the xylan synthase complex is still not completely resolved. The emerging consensus is that IRX10 and related proteins have xylosyltransferase activity (Urbanowicz et al. 2014; Jensen et al. 2018) and provide the active site that links together the xylosyl resides in the backbone. Most, or maybe all, IRX10 and related proteins lack a transmembrane domain. They are thought to associate with the IRX9 and/or IRX14 proteins, which are type II integral Golgi membrane proteins with a single transmembrane domain. It is not yet clear whether the family GT43 proteins have xylosyltransferase activity or whether they have other functions in the xylan synthase complex. Various members of these protein families are utilized depending on the plant species, the plant tissue, and whether primary or secondary wall xylan is being synthesized (Smith et al. 2017; Ye and Zhong 2022; Anders et al. 2023). Anders et al. (2023) postulate that different members of the gene families are involved in xylan synthesis in PCWs than those in SCWs, similar to the situation with the *CESA* family members.

In addition to the various proteins involved in backbone synthesis, Golgi membranes contain several different GTs involved in adding the side chains found on xylans. These include glucuronosyltransferases in CAZy family GT8 (Mortimer et al. 2010; Oikawa et al. 2010) and arabinosyltransferases in GT61 (Ye and Zhong 2022). Because xylans, especially those in SCWs, are heavily acetylated, Golgi membranes also contain several different enzymes, including acetyltransferases, involved in xylan acetyl esterification as well as acetyl esterification of other matrix polysaccharides (Smith et al. 2017; Pauly and Ramirez 2018; Julian and Zabotina 2022), including pectins as described in the next section.

### Pectins

#### Structures of pectins

##### History of pectin and its complexity

Pectin (*pectique*), from the Greek πηκτικοί (to coagulate), was first used by Henri Braconnot to describe the acidic viscous substances with gelling properties isolated from multiple tissues of more than 15 plant species (Braconnot 1825a, 1825b; Wisniak 2007). Braconnot noted that the ability of a very small amount of pectin to gelatinize a large mass of sugar water provided it industrial uses such as in the confectionary business (Braconnot 1825b; Wisniak 2007). These properties led to the establishment of the pectin industry in the early 1900s (Kertesz 1951), and numerous studies on the chemical basis of the gelling properties of pectin from commercial sources such as citrus and apple fruit followed. Braconnot also recognized that pectin's ability to bind and remove toxic metals imparted medicinal properties (Braconnot 1825a), and increasing numbers of industrial, food, and medical applications for pectin have since been explored, including its use in edible coatings on foods, antimicrobial bio-based films, nanoparticles, and as healing agents and cancer treatments (Willats et al. 2006; Freitas et al. 2021). The diverse functional characteristics of pectins led Braconnot to propose that a substance so “universally distributed in plants” “must play a role of great importance in the plant and should be studied by plant physiologists” (Braconnot 1825b, p178). Since then, plant biologists and chemists have confirmed multiple functions for this most structurally complex of the plant cell wall glycans, including roles in plant growth and cell expansion (Proseus and Boyer 2012a, 2012b; Daher et al. 2018; Haas et al. 2020), cell shape (Amsbury et al. 2016; Haas et al. 2020; Lin et al. 2022b), organ size and texture (Zhang et al. 2021a), cell-to-cell adhesion (Marry et al. 2006; Yang et al. 2020a), cell wall porosity (Baron-Epel et al. 1988; Fleischer et al. 1999), cell wall structure (Pérez García 2011), signaling (Feng et al. 2018; Lin et al. 2022b; Du et al. 2022), fruit ripening (De Vries et al. 1981; Paniagua et al. 2014), organ abscission (Daher and Braybrook 2015), pollen development (Mollet et al. 2013), and plant defense (Bacete et al. 2018; Molina et al. 2021; Liu et al. 2023). How does a single class of cell wall glycan provide such diverse functions? This question continues to beset plant biologists.

Understanding pectins’ multiple functions requires knowledge of (1) their primary, secondary, tertiary, and quaternary structure (Diener et al. 2019); (2) covalent and noncovalent interactions between individual pectic polymers and between pectins and other wall polymers; (3) pectins’ roles in cell type-specific wall architecture; and (4) the specific pectic polymeric structure associated with each functional response. In no case is this information currently known. While we know a great deal about the different general types of pectic glycans present in cell wall extracts from different tissues (reviewed by Mohnen et al. 2024), we lack information about the number and structure of pectic polymers in different specific cell types and how they are integrated into cell wall architecture. Furthermore, pectins that are synthesized and inserted into the wall are subsequently modified during development and in response to biotic and abiotic stress by large families of pectin-modifying enzymes, including pectin hydrolases (Yang et al. 2018), lyases (Molina-Hidalgo et al. 2013; Leng et al. 2017), methylesterases (Willats et al. 2006; Wolf et al. 2009), acetylesterases (Philippe et al. 2017), and proteins that modify or inhibit these enzymes (Federici et al. 2006; Hocq et al. 2017b). Importantly, the different classes of enzymes include proteins that uniquely target the different pectic backbones homogalacturonan (HG) and rhamnogalacturonan (see below).

##### The complexity of pectin structure

**Figure 5.**
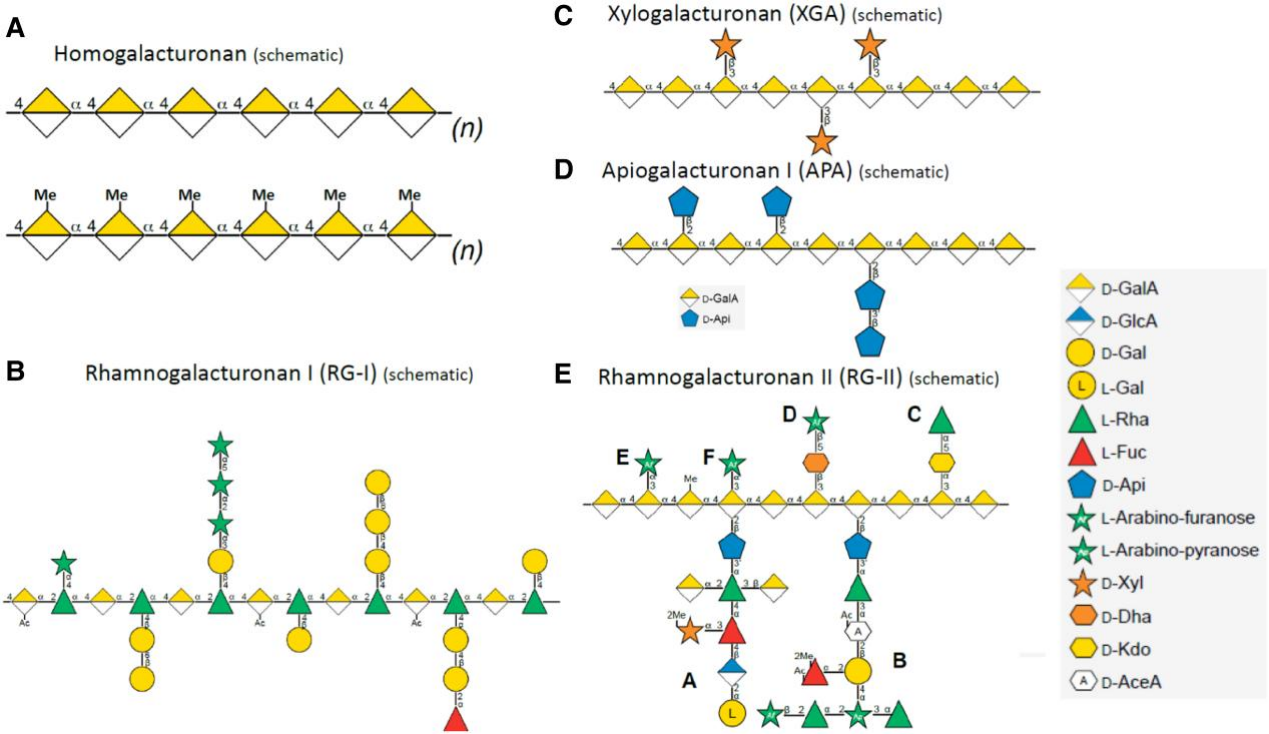
Glycan symbol structure representations of the main pectic glycan domains. **A)** HG. **B)** RG-I. **C)** XGA. **D)** APA. **E)** RG-II. The structures were drawn using Symbol Nomenclature for Glycans from (SNFG) https://www.ncbi.nlm.nih.gov/glycans/snfg.html#nomn.

All pectic polymers have 1 or 2 backbones: HG and/or rhamnogalacturonan. HG is a homopolymer of α-D-1,4–linked galacturonic acid that may have low, partial, or almost full methyl-esterification of the C-6 carboxyl (Bosch and Hepler 2005) (Fig. 5A). It has been proposed that in vivo HG is synthesized in a highly methyl-esterified form; however, the extent and mechanism of esterification of HG synthesized by the different HG biosynthetic enzymes in vivo remain to be determined. HG biosynthetic GALACTURONOSYLTRANSFERASES (GAUTs) can synthesize polymeric HG with no methyl-esterification in vitro (Amos et al. 2018; Engle et al. 2022), indicating that HG methyl-esterification is not a requirement for enzymatic synthesis of HG in vitro (reviewed by Mohnen et al. 2024). Yet, many biological effects are associated with the methyl-esterification status of pectins, and immunolabeling studies indicate that many cell walls have high or changing levels of HG methylester content during development (Wolf et al. 2009).

Each GalA residue in HG has a carboxylic acid group at the C6 position that will be highly ionized and negatively charged at pH above 3.5. Thus, polymeric HG is a polyanion and can interact with positive ions such as calcium or positive patches on proteins. Methylesterification of the carboxyl group by pectin methyltransferases (PMTs) reduces the charge and increases the hydrophobicity of each GalA residue and of methylesterified regions of HG. De-esterification of HG has been associated with changes in wall structure as regions of non-esterified HG can interact to yield HG-Ca^++^-HG crosslinks and such cross-linking can affect the structure and conformation of the HG polymer and of the pectin network in the wall. An understanding of HG structure during development will require knowledge of the extent and pattern of methyl (and acetyl) esterification in different HG-containing polymers as they are inserted into the wall and subsequent modification of HG once it is deposited in the wall. The degree and pattern of HG esterification and HG size in the wall is dependent on cell type, developmental stage, and status of biotic or abiotic stress because these affect the expression of HG degradative and modifying enzymes. These HG-modifying enzymes include pectin methylesterases (PMEs) (Pelloux et al. 2007), PME inhibitors (PMEIs) (Coculo and Lionetti 2022), acetyl esterases (Sénéchal et al. 2014), and polygalacturonases (Kim et al. 2006), which hydrolyze the methyl/acetyl esters and/or the HG backbone, respectively (Amsbury et al. 2016; Liu et al. 2023). Changes in HG methyl esterification and/or HG size are associated with numerous HG functions, including cell:cell adhesion (Kohorn et al. 2021a), cell elongation/shape/growth (Daher et al. 2018; Haas et al. 2020), and fruit ripening (Paniagua et al. 2014).

**Figure 6.**
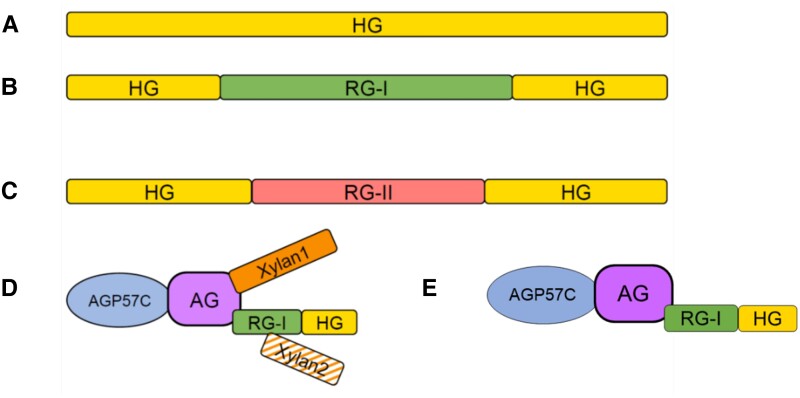
Examples of structurally confirmed homoglycans, heteroglycans, and glycoconjugates that contain the different pectic glycan regions. **A)** HG. **B)** The heteroglycan HG-RG-I-HG. **C)** The heteroglycan HG-RG-II-HG. **D)** The proteoglycan pectic AGP APAP1. **E)** The proteoglycan pectic AGP AGP-RG-I.

There is no definitive evidence for pure HG as a separate polymer in plant cell walls, although pure HG is synthesized in vitro (Fig. 6A) (Sterling et al. 2006; Atmodjo et al. 2011; Amos et al. 2018; Engle et al. 2022). Rather, HG is generally isolated covalently attached to RG-I (Fig. 6B) or to the substituted HGs rhamnogalacturonan II (RG-II) (Fig. 6E), xylogalacturonan (XGA) (Fig. 6C), and apiogalacturonan (Fig. 5D). All substituted HGs have an HG backbone and the requirement for endopolygalacturonase (EPGase) digestion of cell walls to release RG-II and RG-I from the walls has often been interpreted as evidence that RG-II and RG-I exist within an HG polymer and possibly as 1 covalently interconnected heteropolymer in the wall. However, this has not been structurally confirmed, and the architecture of pectins and the different pectic heteroglycans and glycoconjugates in the wall remains a critical question.

RG-II (Fig. 5E) is the most structurally complex of the pectic polymers, with 13 different sugars (Darvill et al. 1978; Kobayashi et al. 1996; Bar-Peled et al. 2012; Ndeh et al. 2017) organized into 6 highly conserved side branches on an HG backbone. Why do plants spend so much energy to make this complex glycan? At least part of the answer is likely the structural role it played during the transition of plants from an aqueous environment onto land (Matsunaga et al. 2004), including wall strengthening via RG-II dimers formed by borate diester complex formation between the side chain A apiosyl residues (Kobayashi et al. 1996; O’Neill et al. 1996; Begum and Fry 2022). Indeed, the dwarf (Reuhs et al. 2004) and cell wall structural (Shi et al. 2017) phenotypes of RG-II mutants support a necessary role for RG-II in cell wall architecture and cell/plant growth (O’Neill et al. 2001).

RG-I is the only pectic polymer not built on an HG backbone (Fig. 6B). RG-I has a GalA-Rha disaccharide repeat backbone [−α−1,4-D-GalA-α−1,2-L-Rha-]_(n)_ with about one-half of the rhamnose backbone residues substituted by single or branched galactose, arabinose, or other sugars (Lau et al. 1985) (Fig. 5B). The degree and type of branching is tissue and developmental stage specific (Kaczmarska et al. 2022, 2023). For example, Arabidopsis seed mucilage RG-I is unbranched (Fabrissin et al. 2019; McGee et al. 2021), whereas RG-I in pea cotyledons is initially arabinosylated and acquires significant β-1,4-galactan side branching only in late development (McCartney et al. 2000). The precise order and location of the side branches is not known for any of the pectic polymers, a point that should be kept in mind when viewing schematic representations of pectic heteroglycan structures.

##### Conformation of pectins

Structure dictates function. Beyond pectins’ primary structures (which are already very complex), their secondary (e.g. degree and type of helix formation), tertiary (single molecule 3D structure), and quaternary (polymer-polymer association) structures lead to their vast diversity of biological and industrial functions. Yet, our understanding of these higher levels of pectin structure is unclear (Zdunek et al. 2021). In the mid-1930s, Bonner concluded—based on X-ray diffraction and viscometry studies—that pectins existed as long chains (Bonner 1936). It was known that pectins isolated from plants ranged from high to low methyl esterification content and that the amount of free carboxyl groups affected pectins’ colloidal properties (Bonner 1936). Subsequent x-ray diffraction analysis of isolated methyl esterified and de-esterified pectate in the absence and presence of calcium revealed a fibrillar 3_1_ (3 residues per turn) helical quaternary conformation for various forms of HG, including sodium pectate, pectic acid, methylesterified HG (pectinic acid), and calcium salt–bridged HG (Walkinshaw and Arnott 1981a, 1981b). Shortly thereafter, Rees and colleagues presented evidence for a 2_1_ conformation (2 residues per turn) of calcium pectate dimers, naming this conformation the egg box structure (Morris et al. 1982). More recent modeling suggests further conformational complexity (Braccini and Pérez 2001; Pérez et al. 2003; Braccini et al 2005), and the conformation of HG in vivo remains a matter of debate. Regarding the substituted HG RG-II, Pérez and colleagues provided evidence for a compact, flat, disc-like RG-II monomer structure with compaction of each monomer during dimerization yielding RG-II dimer conformations akin to 2 flattened, parallel disks stacked on top of each other (Pérez et al. 2003). Recent studies of RG-I backbone oligosaccharides indicated a 3_1_ right-handed helical structure with 2 backbone disaccharide repeats per turn (Scanlan et al. 2022). Clearly, future studies to clarify the in vivo and in vitro conformation(s) of pectins are critical to delineate pectin structure/function relationships.

**Figure 7.**
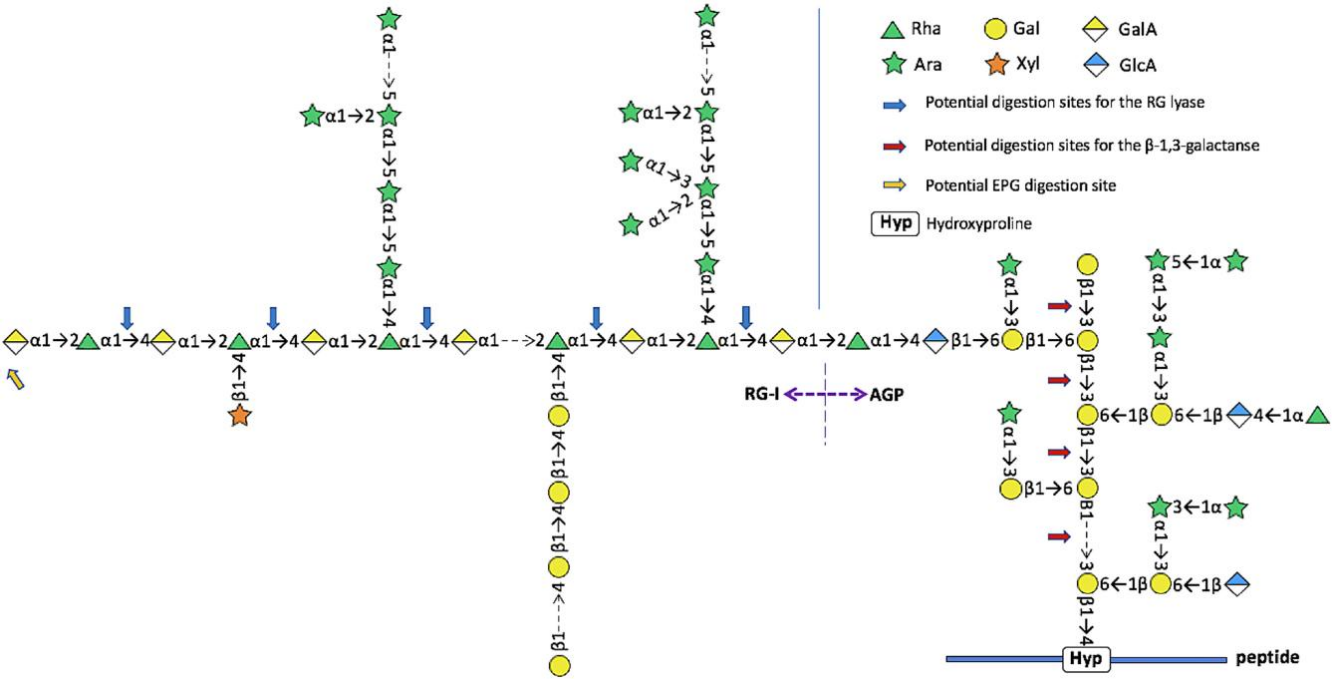
Glycan symbol structure representation of pectic AGP isolated from cell walls of Arabidopsis suspension culture cells. Pectic AGP RG-I-AGP from suspension culture cell walls (from Tan et al. 2023b). The structures were drawn using Symbol Nomenclature for Glycans from (SNFG) as described in Fig. 5.

Numerous reports over many years have indicated that pectins may be attached to proteins or hemicelluloses (reviewed in Mohnen et al. 2024). Selvendran (1985) presented evidence for pectin-hydroxyproline and serine-rich protein complexes in cell walls of parenchymatous tissues from multiple species. Later, Mort and colleagues reported pectin-protein cross-links in cotton that appeared to be between RG-I and extensin (Qi et al. 1995). The identification of a covalent connection between the arabinogalactan domain of AGP57C and the backbone of RG-I in the pectic AGP APAP1 (arabinoxylan pectin arabinogalactan protein 1) isolated from the medium of Arabidopsis suspension cultures (Tan et al. 2013) provided a biochemical confirmation of a covalent linkage between pectins and protein. APAP1 had short xylan chains linked to the arabinogalactan domain and to Rha in the RG-I backbone. The RG-I backbone was elongated on the nonreducing end with HG (Fig. 6, D and E). Importantly, in a recent study of RG-I isolated from the cell walls of Arabidopsis suspension cells, a pectic AGP named RG-I-AGP was identified as the most abundant form of RG-I present in those walls (Tan et al. 2023b). Because the pectic AGP was isolated by the classical EPGase digestion of the cell walls followed by size exclusion chromatography, the results indicated that the bulk of classically defined RG-I in the walls was covalently linked to protein (Tan et al. 2023b) (Fig. 7). The RG-I-AGP was similar to APAP1; however, it did not have covalently linked xylan chains but rather single xylose residues attached to the RG-I backbone. Efforts to delineate whether all RG-I is synthesized as pectic AGP and, if so, how much is connected to the plasma membrane via GPI-anchoring of the AGP protein core versus how much is synthesized as a free heteroglycan not attached to protein [e.g. existing between HG domains in an HG-RG-HG glycan (Nakamura et al. 2002)] is critical to understanding pectin structure and cell wall architecture. It is noteworthy that the negatively charged family of pectic heteroglycans and glycoconjugates have structural and functional analogies to animal glycosaminoglycans and proteoglycans (Varki et al. 2022).

#### Biosynthesis of pectins

HG is synthesized by the CAZy GT8 GAUT family in Arabidopsis with GAUTs 1, 4, 10, 11, 13, 14, and possibly more functioning as catalytic HG biosynthetic GAUTs and 3 (GAUTs 5, 6, 7) functioning as Golgi-anchoring subunits of GAUT1 in GAUT1 protein complexes (Lund et al. 2020; Engle et al. 2022). All catalytic GAUTs synthesize polymeric HG in vitro by acceptor-dependent mechanisms, but some such as GAUTs 13, 14, and 1 can also de novo synthesize HG using only UDP-GalA as both the donor and acceptor substrate (Sterling et al. 2006; Atmodjo et al. 2011; Amos et al. 2018; Engle et al. 2022). The role of GAUT4 in wood formation in trees and secondary wall formation in grasses was probed due to its high expression levels in switchgrass (*Panicun virgatum*) stems and during transition from primary wall to wood formation in poplar (*Populus trichocarpa*). Downregulation of GAUT4 in switchgrass, poplar, and rice resulted in greatly reduced amounts of HG and RG-II (Biswal et al. 2018), indicating that in herbaceous dicots, grasses, and trees, GAUT4 may synthesize the bulk of HG and the HG backbone for RG-II. These results provide support for the existence of heteroglycan HG-RG-II-HG glycans (Fig. 6C). Interestingly, there was no reduction in RG-I in these plants, indicating that the synthesis of RG-I is not dependent on the synthesis of the bulk of HG, nor on the synthesis of RG-II. Interrogation of GAUT4 activity in a species with a single GAUT4 homolog, such as Arabidopsis, is challenging due to apparent lethality upon loss of gene function (Caffall et al. 2009). However, expansion of the GAUT family in poplar and switchgrass to 2 and 6 GAUT4 homologs, respectively, allowed knockdown expression of the most highly expressed GAUT4 homolog and analysis of phenotypes. The increase in cell size in the GAUT4 knockdown plants (Biswal et al. 2018) supports proposed roles for the pectin cross-linked matrix in plant cell growth and expansion (Proseus and Boyer 2012a, 2012b; Haas et al. 2020, 2021) (see Models of Cell Wall Organization section below).

Progress has been made in identifying some enzymes involved in synthesis of the substituted HGs. Xylogalacturonan Deficient 1 was identified as a GT47 xylosyltransferase that adds β-Xyl onto the 3-position of GalA in the HG backbone (Jensen et al. 2008), providing tools to explore the biological significance of XGA substitution within the HG backbone. Although originally believed to be most prevalent in fruits, XGA has been identified in many cell types (Zandleven et al. 2007). Immunological evidence suggests it may be associated with plant cell separation and detachment (Willats et al. 2004); however, a complete characterization of the epitope recognized by the antibody will be required to substantiate the functional interpretation of the immunolabeling results, a caution associated with many immunolabeling results. Little is known about the synthesis of the most universal substituted HG, RG-II. Three xylosyltransferases that add α-xylose to the 3-position of L-Fuc (RGXT) (GT77) in RG-II chain A have been identified (Egelund et al. 2006, 2008), but genes encoding the other RG-II biosynthetic enzymes remain to be identified.

The methylesterification state of HG dramatically affects its in vivo and industrial properties, with the pattern and degree of esterification affecting pectins’ gelling properties, ability to form pectate calcium ionic crosslinks, and sensitivity to enzymatic cleavage by polygalacturonases (Willats et al. 2006; Wolf et al. 2009). Correspondingly, considerable effort has gone into searches for the methyltransferases that esterify the HG backbone. However, although many mutants affected in methyl ester content have been identified, only with the recent biochemical confirmation of the HG methyltransferase activity of *Quasimodo2* has the door been opened to understanding this crucial process (Du et al. 2020a). Critical experiments for the future include determining whether methyltransferases function in complexes with HG biosynthetic enzymes and the mechanisms for HG methyl esterification levels and patterning during synthesis.

The identification of the biosynthetic RG-I backbone rhamnosyltransferase (RRT) (GT106) (Takenaka et al. 2018) and galacturonosyltransferase (RGGAT) (GT116) (Amos et al. 2022) families that function together to synthesize the RG-I rhamnogalacturonan backbone (Fig. 6B) opens the door to in vitro synthesis of complex pectic heteroglycans and pectic AGP glycoconjugates such as APAP1 (Tan et al. 2013) and RG-I-AGP (Tan et al. 2023b) (Fig. 7D), as well as to efforts to reconstitute the galactose- and arabinose-rich side chains.

Complex RG-I synthesis will require identification of the enzymes that initiate side branch formation on the backbone, enzymes that thus far have remained elusive. The identification of the β-1,4 galactan synthases (GALS) (GT92) that extend the galactose side branches (Liwanag et al. 2012; Ebert et al. 2018; Laursen et al. 2018) was an important step forward and provided catalysts for study of type II galactan synthesis and structure. Interestingly, GALS1 was demonstrated to be a bifunctional enzyme that also can add arabinose to β-1,4-galactan chains, an activity hypothesized to function in chain termination (Laursen et al. 2018; Prabhakar et al. 2023). The 3D crystal structure of GalS1 from *P. trichocarpa* revealed a modular protein structure with an N-terminal CBM95 RG-I backbone binding domain, C-terminal GT92 GT-A fold catalytic domain, and stem region involved in homodimer formation (Prabhakar et al. 2023). Mutagenesis informed by comparison of GT-A fold sequences to the GT92 family identified a novel histidine as the catalytic base and other amino acids involved in β-1,4-galactan synthesis and acceptor binding. The approaches used in this study, including docking with molecular dynamics simulations, exemplify structure-function and acceptor specificity tools that may accelerate future progress on identifying and characterizing the enzymes involved in plant cell wall synthesis.

The combination of genetic loss-of-function phenotype with enzyme activity determined by in vitro heterologous expression of encoded protein sequence is the most definitive evidence for biochemical function of cell wall GTs (Amos and Mohnen 2019). Two putative arabinosyltransferases (ARAD1 and ARAD2) (GT47) that may add α−1,5-linked Ara onto RG-I side branches have been identified via studies of the *arabinan deficient 1* mutant and molecular complementation (Harholt et al. 2006, 2012). Although biochemical confirmation of enzymatic activity has not yet been provided, these enzymes provide tools to attempt in vitro reconstitution of complex RG-I via coexpression with RG-I backbone synthesizing enzymes.

The identification of pectin biosynthetic genes has enabled the generation of cell wall glycan–modified mutant/variants and their probing using nanoscale imaging to test the hypothesis that pectins interact with cellulose and that hemicelluloses and pectins share load-bearing functions in the wall (Pérez García 2011). Coexpression in Arabidopsis stems of cytosolic UDP-Glc epimerase, Golgi localized UDP-Rha/UDP-Gal transporter, and RG-I GALS1 increased RG-I galactan content by 50% (Aznar et al. 2018). Multidimensional solid-state NMR analysis of C^13^-labeled high galactan-expressing Arabidopsis stems showed an increased proximity between galactan and cellulose in the secondary walls, leading the authors to hypothesize that increased galactan chain lengths led to increased mobile contacts between galactan and cellulose and longer spatial contacts between xylan and pectins in Arabidopsis SCWs (Gao et al. 2023). More research is required to understand the degree and importance of molecular interactions between pectins and cellulose in the cell wall, critical information for generation of meaningful cell wall models (see below).

#### Functions of pectins in vivo

##### Hypothetical model for pectin structure in the wall

A biologically meaningful interpretation of pectin mutant phenotypes requires, at minimum, knowing how many different types of pectic polymers exist in the wall and how they are covalently and noncovalently connected to each other and to other polymers. At present, pectic polymer models do not provide this information. Thus, a conundrum confronts us. On the one hand, making a model that is structurally inaccurate can lead to erroneous molecular genetic conclusions. On the other hand, without a model, functional hypotheses cannot be made.

Testing hypotheses of pectin function requires knowing which pectic glycan (Fig. 6A), heteroglycan (Fig. 6, B and C), or glycoconjugate (Fig. 6, D and E) is affected in the mutant/variant of interest. For example, the homogalacturon structure depicted in Fig. 6A has been shown to be synthesized in vitro as a nonmethylesterified homopolymer by GAUTs 10, 11, 13, 14, and the GAUT1:7 complex (Amos et al. 2018; Engle et al. 2022; Mohnen et al. 2024), and yet, there is scant evidence that pure homogalacturon exists as an isolated polysaccharide in the wall.

**Figure 8.**
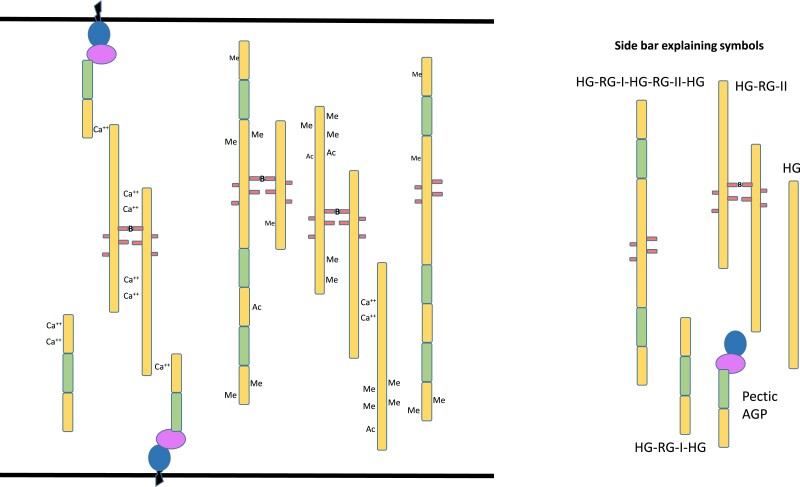
Pectic heteroglycan and glycoconjugate model. In this testable hypothetical model, pectin in the primary wall is proposed to consist of 5 different pectic polymeric structures: a small amount of HG, the pectic heteroglycan HG-RG-II-HG; the pectic heteroglycan HG-RG-I-HG, the pectic glycoconjugate pectic-AGP, and the possible complex pectic heteroglycan HG-RG-I-HG-RG-II-HG representing the covalent connection of the separate glycan and heteroglycan polymers. These 5 pectic polymers may interact via ionic interactions and covalent boron diester cross-linking. Representative methyl and acetyl esterification of the HG backbone is shown but no specific pattern is implied. No side glycan or single sugar side branching is shown but is implied as in Fig. 5. RG-II dimers could occur between any 2 adjacent RG-II molecules. The model is not meant to be quantitatively accurate but rather to denote possible heteroglycan and glycoconjugate structures.

To develop a more pectin polymer-based vocabulary for discussing pectin structures and pectin mutant phenotypes, a working hypothetical model for pectin polymeric structures in plant cell walls is proposed. The pectic heteroglycan and glycoconjugate model (Fig. 8) depicts pectins as consisting of 5 different possible glycans, heteroglycans, and glycoconjguates. These include the covalently linked pectic heteroglycan HG:RG-I-HG-RG-II-HG, the separate heteroglycans HG-RG-I-HG and HG-RG-II-HG, the glycan HG by itself, and the pectic glycoconjugate pectic-AGP. The pectic polymers interact via ionic HG-calcium salt bridges and covalent RG-II borate diester cross-links. The pectic AGP is bound to the plasma membrane via a GPI anchor on the AGP protein core (although cleavage by phosphodiesterases may solubilize this into the wall). Representative methyl and acetyl esterification of the HG backbone is shown, but no specific pattern is implied. It is likely that the degree and pattern of methyl and acetyl esterification is development-, tissue-, cell-type-, and stress-level dependent. This model is supported by the observation that RG-I and RG-II are released from cell walls by treatment with EPGase and the supposition that although it is possible that they may all be covalently connected in a single network, it is also possible that they exist as separate heteropolymers in the wall (reviewed in Mohnen et al. 2024). Some of the RG-I and HG are also depicted as pectic AGPs due to the observation that RG-I in Arabidopsis exists in this form (Tan et al. 2013, Tan et al. 2023b). The backbones of the polymers are depicted, but it is understood that RG-I will have diverse side chains depending on the tissue and developmental stage. Future studies of pectin synthesis and mutant phenotypes are required to test the hypothesized pectic polymeric structures.

##### Pectin location

Plant biologists soon recognized that pectins were the main components in the middle lamella between cells and were also present in the PCW, along with cellulose, hemicelluloses, and to a lesser extent lignin (Mangin 1893a, 1893b; Kerr and Bailey 1934). Some, including Mangin (1893b) and Bonner (1936), suggested that pectins in the middle lamella existed as calcium pectate, whereas others concluded that they existed as “protopectin” and/or a pectin-protein complex (Sloep 1928; Kertesz 1951). More recent results with antibodies recognizing RG-I (Moore and Staehelin 1988) and transgenic plants expressing reduced levels of pectin metabolic enzymes clearly show that the pectin in the middle lamella contains not only HG but also RG-I (Molina-Hidalgo et al. 2013).

**Figure 9.**
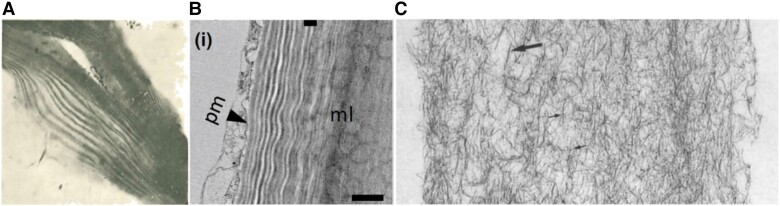
Microscopic evidence for alternating pectin-cellulose layers in collenchyma cells and fibrillary nature of pectin. **A)** A single tomato collenchyma cell wall after treatment with potassium iodide and sulfuric acid to remove pectin and expose cellulose lamellae, ×300 magnification (from Fig. 10 from Anderson 1927). **B)** TEM images of longitudinal sections of celery petiole collenchyma cells showing lamellae of cellulose MFs stained with uranyl acetate and lead citrate. Pm: plasma membrane (from Fig. 1i from Chen et al. 2019). **C)** Fast-freeze, deep-etch rotary shadowed (FDR) replica image of tomato suspension cells adapted to growth on 12 *µ*M DCB showing pectin fibers in wall (from Fig. 3E from Wells et al. 1994).

Although less appreciated, considerable data indicate a function for pectins in walls that span the primary-secondary wall definition. There are multiple examples of alternating layers of pectins and cellulose in so-called collenchymatous secondary walls (Anderson 1927; Bonner 1936; Chen et al. 2019), a location consistent with GAUT1 expression in both primary and secondary (xylem) walls in Arabidopsis (Atmodjo et al. 2011). A microscopic study of freshly cut tomato (*Solanum lycopersicum*) (Anderson 1927) provided early evidence for a lamellar wall structure and cellulose-pectin interactions, including alternating layers of protopectin and cellulose in collenchymatic secondary walls (Fig. 9A), a finding confirmed in celery collenchyma walls, walls defined by the authors as thickened PCWs because the cells were still elongating (Chen et al. 2019). Solid-state NMR revealed reduced mobility for pectins and cellulose as the walls matured and expansion ceased (Chen et al. 2019) (Fig. 9B).

It has been proposed that the specialized secondary walls in fibers in G layers of tension wood, extraxylary flax (*Linum usitatissimum*), and hemp (*Cannabis sativa*) fibers, and fibers of climbing plants are different from other secondary walls in that they have a very high cellulose content (85% to 90%), a very low amount of lignin and xylan, and the presence of a unique RG-I highly substituted with galactan (Gorshkova et al. 2022). In such fibers, RG-I is first synthesized with long galactan side chains and inserted between cellulose MFs, preventing lateral association of neighboring MFs. Over time, β-1,4-galactosidase cleaves the galactan chains and RG-I lyase cleaves the backbone, enabling lateral association of the cellulose fibrils and leading to tension that can result in contractile properties of the fibers. These results indicate that pectins have roles in unique secondary walls and in promoting stems to bend and climb.

##### Pectins in cell:cell adhesion and fruit ripening

Fremy (1847, 1848) proposed the existence of pectose/protopectin in vegetables, fruits, and roots as an insoluble pectin that could be converted into the soluble pectins (reviewed in Bonner 1936) and concluded that what most people studied as pectin was a portion of the original protopectin in plant tissues. Fremy (1848) and Carre (1922) also attributed the thinning of fruit cell walls during ripening to the “decomposition of the insoluble protopectin and the formation of soluble pectin” (Bonner 1936; Kertesz 1951), and enzymes were identified that catalyzed such degradation. Thus, already in the 1800s and early 1900s, functions of pectins were recognized in cell:cell adhesion and fruit texture, and it was understood that easily extractable pectins represented only a portion of pectins in the wall. Both HG (Shedletzsky et al. 1992; Liners et al. 1994; Sobry et al. 2005) and RG-I (Yang et al. 2020a) have since been confirmed to function in cell:cell adhesion, leading to the question of whether they function together in a given cell type or have unique roles in different cell types and tissues. The differential abundance of HG and RG-I in growing vs nongrowing cells, respectively, would support the latter (Mohnen et al. 2024).

##### Pectin fibers

For more than 50 years, extensive evidence has indicated the existence of pectin fibrils or filaments, yet most modern models of pectins do not represent such structures. In 1951, Roelofsen and Kreger showed, using polarization microscopy, diffraction, and electron microscopy, that pectins in collenchyma cell walls of butterbur (*Petasites hybridus*) existed as oriented fibrils and not, as had been previously held, as amorphous and optically isotropic (Roelofsen and Kreger 1951). Staehelin and Pickett-Heaps (1975) reported pectinase-sensitive fibrils with a 3- to 13-nm diameter in the Chlorophyceaen green alga Scenedesmus. Shortly thereafter, Leppard and Colvin (1972) identified electron opaque ruthenium red-staining pectin fibrils in root epidermal cells. More recently, pectic nanofibers have been demonstrated in Arabidopsis epidermal pavement cells, and roles for the fibers in cell expansion have been proposed (Haas et al. 2020). The evidence for pectic fibers highlights the question of the tertiary and quaternary structure of pectins, and more specifically HG. As there is increasing evidence for pectins associating with and between cellulose MFs and fibers (Zykwinska et al. 2005; Lin et al. 2016), the importance of understanding pectin structure and architecture in the wall and in association with cellulose is increasingly important. Recently, resonant X-ray scattering of onion epidermal cell walls revealed a 20-nm spacing of cellulose MFs with a pectin matrix between the fibrils (Ye et al. 2018).

##### Pectins in cell growth and expansion

Tomato cells adapted to growth on DCB are able to divide and expand with a “virtual absence of a cellulose-xyloglucan network” (Shedletzky et al. 1990). Subsequent studies of the DCB-adapted cells revealed enhanced wall-membrane connections and an accompanying reduced ability to plasmolyze the cells, an ability that could be recovered by treatment of the cells with 50 mm CDTA and a pure endopolygalacturonase. These results implicated calcium-homogalacturonan salt bridges as load-bearing components that could support growth of the DCB-treated cells (Shedletzky et al. 1992). Imaging of the DCB-adapted cells provided a visualization of a fibrillar pectin network in the absence of the cellulose-xyloglucan network (Wells et al. 1994) (Fig. 9C). The pectin load-bearing hypothesis was supported by lysis of tobacco and tomato DCB-adapted cells by overnight treatment with CDTA, a lysis that did not occur in non-adapted cells. Although the tensile strength of the DCB-adapted cells was less than one-half that of normal cells, as expected due to the drastic reduction in cellulose, the ability of the calcium cross-linked pectate (i.e. HG) to provide sufficient load-bearing to keep the cell intact and enable growth provided strong evidence for a role of a pectin cross-linked matrix in cell expansion and growth in dicot cells (Shedletzky et al 1992). Further evidence of a role for pectins in cell expansion is presented below (see Models of Cell Wall Organization section).

### Wall-associated proteins

#### Types of cell wall proteins

Plant cell walls contain a diverse collection of proteins, glycoproteins, and proteoglycans that perform an array of different functions (Albersheim et al. 2011). These can be organized into 2 general classes, insoluble and soluble wall proteins, with each class further subdivided into multiple categories (Albersheim et al. 2011). The insoluble proteins are tightly bound to the cell wall and are generally considered to play structural roles; these include large families of hydroxyproline-rich proteins (HRGPs), extensins (EXTs), proline-rich proteins (PRPs), and glycine-rich proteins (Dougall and Shimbayashi 1960; Lamport and Northcote 1960) and have been extensively reviewed (Varner and Lin 1989; Showalter 1993; Kieliszewski and Lamport 1994; Ringli et al. 2001; Showalter et al. 2010; Lamport et al. 2011; Moussu and Ingram 2023). The soluble proteins include enzymes, lectins, transport- and defense-related proteins, as well as hydroxyproline-rich arabinogalactan proteins (AGPs) (Silva et al. 2020). The AGPs may be insoluble or soluble, depending on whether they are covalently attached to wall polysaccharides (reviewed in Tan et al. 2023a, 2023b). Cell wall enzymes include glycan exo- and endo-hydrolases and lyases, transglycosidases, methyl and acetyl esterases, peroxidases and laccases involved in lignin polymerization, enzyme inhibitors, lipid transport proteins functioning in cutin and wax synthesis, and proteases.

Another way to classify the large superfamily of plant cell wall HRGP proteins is via their level of glycosylation, with AGPs being hyperglycosylated, extensins moderately glycosylated, and PRPs lightly glycosylated. The AGP proteins contain noncontiguous Hyp residues and are about 90% carbohydrate by mass, while the extensins have regions of contiguous Hyp residues such as Ser-Pro-Pro-Pro-Pro motifs that become hydroxylated and arabinosylated with 1 to 4 Ara*f* residues (Kieliszewski et al 1992a, 1992b; Petersen et al. 2021). The “Hyp-contiguity hypothesis” developed by Kieliszewski and Lamport (1994) proposes that contiguous Hyp residues [Ser-Hyp4] (as found in extensins) are sites for short arabinose oligosaccharide addition, whereas repetitive noncontiguous Hyp residues (as found in AGPs) are sites for Type II arabinogalactan attachment.

Because it is difficult to identify the large classes of insoluble proteins by BLAST analyses of amino acid sequence due to overlapping protein sequence motifs and arrangements, Showalter et al. (2010) developed a multifaceted bioinformatics approach and identified 166 Arabidopsis HRGPs that grouped into 85 AGPs, 59 EXTs, 18 PRPs, and 4 AGP/EXT hybrids. In poplar, with its expanded genome and large amount of woody secondary walls, a similar approach identified 271 HRGPs, including 162 AGPs, 60 EXTs, and 49 PRPs. The bioinformatics analyses identified and distinguished EXTs with variations of the X-Hyp_4_ sequences including SP3, SP4, and SP5. There are also EXT hybrid proteins with additional domains such as the PERKS (proline-rich extension-like receptor kinases), formins with an EXT motif and actin binding domain, hybrid-EXTs such as the LRR-EXTs (leucine rich repeat EXTs), and hybrid AGP-EXT (Borassi et al. 2016). Many of the prolines in the repetitive domains of HRGPs are hydroxylated, and these may, depending on the repetitive amino acid motif, be heavily or partially glycosylated. Pro-Hyp-Val-Tyr-Lys regions in HRGPs appear to be involved in intermolecular crosslinking, adhesion, and cohesion, and Tyr-X-Tyr-X in isodityrosine and pulcherosine covalent cross-linking and self-assembly catalyzed by class III peroxidases (Kieliszewski and Lamport 1994; Showalter et al. 2010; Mishler-Elmore et al. 2021). Analysis of coexpressed GTs, prolyl 4-hydroxylases, and peroxidases have provided candidate enzymes involved in glycosylation, proline hydroxylation, and crosslinking, respectively, and progress is being made in delineating the biosynthetic pathways for these post-translational modifications that are critical for HRGP function (Silva et al. 2020).

The AGPs are a diverse, highly glycosylated, multi-functional subgroup of HRGPs. Tony Bacic and his colleagues in Australia were major contributors to our understanding of this interesting group of complex glycoproteins (Ellis et al. 2010). They are found across all plant species, including algae, bryophytes, and ferns (Seifert and Roberts 2006; Silva et al. 2020; Ma and Johnson 2023; Tan et al. 2023a). AGPS are classified as classical AGPs, lysine-rich AGPs, arabinogalactan peptides, fasciclin-like AGPs, plastocyanin AGPs, and chimeric AGPs (Showalter et al. 2010; Shafee et al. 2020). Of the 85 AGPs in Arabidopsis, 56 were predicted to have a C-terminal GPI anchor sequence (Showalter et al. 2010) directing them to the outer leaflet of the plasma membrane, where it is hypothesized that they remain tethered unless cleaved from the wall by phospholipases. The term GPI anchor is a misnomer because the plant glycolipid contains an inositol ceramide rather than phosphatidylinositol as found in animals (Silva et al. 2020).

The AGPs are glycosylated on selected Hyp residues by large β-1,3-galactan and β-1,6 branched side chains (Type II arabinogalactans) that can be further decorated with sugars such as Ara*f*, Rha, Gal, Fuc, and GlcA. Some AGPs, such as Arabidopsis AGP57C, also have covalently attached pectic glycan RG-I, which is further elongated with HG producing so-called pectic AGPs, and some of these also contain covalently linked xylan (Tan et al. 2013, Tan et al. 2023b) (see Fig. 6). Classic AGPs can be precipitated with β-Yariv reagent, which has been used to detect AGPs in the wall, but pectic AGPs are not reactive with Yariv reagent due to their complex glycosylation. AGP glycosylation is species and tissue dependent with potential multiple AG chains per protein core and with 65 to 142 sugar residues per AG chain reported (Tan et al. 2023a). Although progress continues in defining specific AGP glycosyl structures, we know very little about the variation of specific AG side chain structures within a given cell type or in different tissues or differences in glycosylation of different AGP protein cores. AGP glycan structural diversity is due to the specificity of different GTs and to any glycan processing by hydrolases that may occur within the Golgi complex, as recently suggested for Golgi localized CAZy GH43 β-1,3-galactosyl hydrolase (Nibbering et al. 2020).

#### Functions of cell wall proteins in vivo

The AGPs are the largest family of cell wall proteins and perhaps the most multi-functional, with roles including plant growth and development, cell expansion, division, signaling, embryogenesis, vascular and gametophyte development, and biotic and abiotic stress response (Seifert and Roberts 2006; Silva et al. 2020; Lin et al. 2022a; Leszczuk et al. 2023; Ma and Johnson 2023; Tan et al. 2023a). Mechanisms for how they may carry out these functions include mediation of information between the cell wall, plasma membrane, and cytoplasm in part due to their anchoring to the plasma membrane and covalent and/or noncovalent interactions with wall carbohydrates and/or proteins. The amount of glucuronidation of the AGP has been associated with calcium binding, signaling, and salt tolerance (Lopez-Hernandez et al. 2020); covalent connection of AGPs to pectin has been implicated in the establishment and maintenance of wall structure and cell expansion (Tan et al. 2013; Biswal et al. 2018; Tan et al. 2023b). AGP signaling mechanisms include indirect signaling through effects on wall properties, action as co-receptors, and activity of the arabinogalactan carbohydrates as signaling molecules (reviewed in Seifert and Roberts 2006; Ma and Johnson 2023). For example, an arabinogalactan 4-methyl-GlcA-β-1,6-Gal terminal AGP epitope from *Torenia fournieri* mature ovules, known as AMOR, has been shown to induce competency of pollen tubes to respond to attractant peptide signals from the synergid cell, thereby identifying specific AGP glycans as signals required for guidance of pollen tubes and pollination (Mizukami et al. 2016), although the specific AGP(s) on which the AG signaling glycan is present remain to be determined. A mechanistic understanding of the roles of AGPs in cell wall structural integrity and cell wall signaling, however, remains fragmentary for most AGPs. A key area of research for the future is to understand how different AGPs and other HRGPs interact in the wall with each other and with carbohydrates, lignin, and proteins, and in those cases where the AGPs are direct signaling molecules to identify their ligand and receptor/co-receptor partners and signaling pathways. Physiological roles for some of the AGPs are just now being identified through molecular genetic analyses of biosynthetic mutants (reviewed in Moreira et al. 2023).

**Figure 10.**
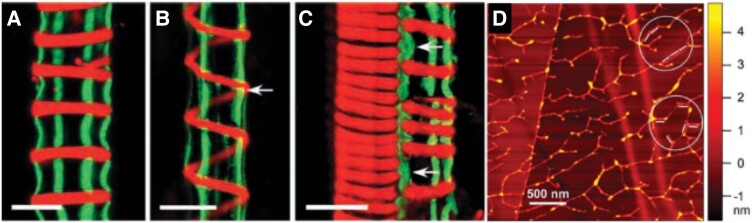
Evidence for roles of structural cell wall proteins in wall structure, assembly, and architecture. **A to C:** Confocal microscopy 3D reconstruction of protoxylem (PX) in apical region of soybean hypocotyl immunolocalized with anti-GRP1.8 polyclonal rabbit antibody (green fluorescence) and lignin (red fluorescence). **A)** PX with ring-shaped lignified SCW and GRP structure. **B)** PX with helical lignified SCW and GRP structure present during passive PX elongation. **C)** Two PXs with younger one (left) with closely spaced SCW and no GRP structure and older one (right) with more widely spaced SCW and GRP structure (taken from Ryser et al. 2003, Fig. 1, A–C). **D)** In vitro self-assembled Arabidopsis EXT3 network imaged by AFM (taken from Cannon et al. 2008, Fig. 5).

As a group, the structural wall proteins are thought to be part of a cell wall network that functions within or is associated with the cellulose-hemicellulose-pectin carbohydrate network, thereby providing or contributing to mechanical strength and possibly to assembly or architecture of the wall (Fig. 10). It has been proposed that EXTs with their positive charge may interact with pectins to form a protein-carbohydrate polymer gel-like matrix and possible scaffold with the potential to respond to pH and ionic conditions in the wall (Tierney and Varner 1987; Lamport et al. 2011). The repetitive motifs and structures of the EXTs (and other HRGPs) certainly support structural roles (Mishler-Elmore et al. 2021), but the difficulty of extracting them from walls due to cross-linking with the protein, carbohydrate, and lignin components has made this difficult to prove (Qi et al. 1995; Nuñez et al. 2009). In no case has a specific structural role of EXTs in the wall been unambiguously confirmed (Moussu and Ingram 2023). However, recently LEUCINE-RICH REPEAT EXTENSIN (LRX8) in pollen tubes was shown to form a complex with the peptide RAPID ALKANIZATION FACTOR 4 (RALF4), thereby exposing a positive patch on RALF4 that bound HG and stabilized a reticulate cell wall architecture (Moussu et al. 2023) that strengthened the pollen tube wall during growth. A similar case for root hairs involving LRX1 and RALF22 is discussed in more detail in the signaling section (Schoenaers et al. 2023).

Transcript expression analyses suggest that most EXTs, about one-half the AGPs, and a few of the PRPs have organ-specific expression, suggesting that some HRGPs may have roles within most cells whereas others have cell-, tissue-, or organ-specific function. Immunolabeling of wall proteins has revealed cell type and sub-cell wall–specific labeling for some HRGPs. For example, the Arabidopsis proline-rich cell wall protein AtPRP3 is preferentially expressed in root hair–bearing epidermal cells at the root/shoot junction and in the root differentiation zone of light-grown seedlings (Bernhardt and Tierney 2000). Molecular genetic analyses support a role for AtPRP3 in cell wall structure in differentiating root hairs (Bernhardt and Tierney 2000; Albersheim et al. 2011).

Other HRGPs are localized to SCW-rich tissues. In etiolated soybean hypocotyls, bean GRP 1.8 localizes to protoxylem, the water-conducting tissue of young and elongating tissues (Keller et al. 1989; Ryser 2003). Deposition of GRP 1.8 begins in cell corners between protoxylem elements and develops further to interconnect secondary walls between adjacent protoxylem and form a GRP structural wall between 2 dead protoxylem cells. It also connects protoxylem elements and xylem parenchyma cells in a 3-dimensional GRP network that stabilizes the whole protoxylem by interconnecting ring and spiral-shaped secondary wall thickenings of the protoxylem and the middle lamella of living xylem parenchyma cells, preventing premature collapse of the protoxylem. Confocal laser scanning microscopy of pectinase-softened hypocotyls using anti-GRP1.8 antibodies identified the GRP structure as a distinct structural element arranged in the longitudinal axis of the protoxylem elements (Fig. 10, A–C) (Ryser et al. 2003) that is proposed to function in stabilizing cell corners and anchoring and stabilizing protoxylem elements (Ryser 2003). The GRP structural element is closely associated with an RG-I and HG pectin network (Ryser 2003; Ryser et al. 2003), providing further evidence for a function of pectins in secondary walls and a role for passive elongation of protoxylem cells (Fig. 10B).

In addition to providing mechanical strength to walls, EXTs are proposed to self-assemble into a network structure that may serve as a positively charged scaffold for pectin in the wall (Fig. 10D) (Cannon et al. 2008) (see “Life of a Cell Wall” below), be involved in wall repair and response to microbial interactions, and be induced when plants are wounded and/or attacked by pathogens (Petersen et al. 2021). Although the name extensin was coined by Lamport (1963) with the proposition that such proteins may be involved in cell expansion, there are many reports in which secretion of extensin into the walls is correlated with the cessation of cell growth (Sadava et al. 1973; Mishler-Elmore et al. 2021). EXTs have also been associated with stresses, including ethylene, wounding, and cold and hot temperatures (Swords and Staehelin 1989).

### Lignin

#### Structure and function of lignin

**Figure 11.**
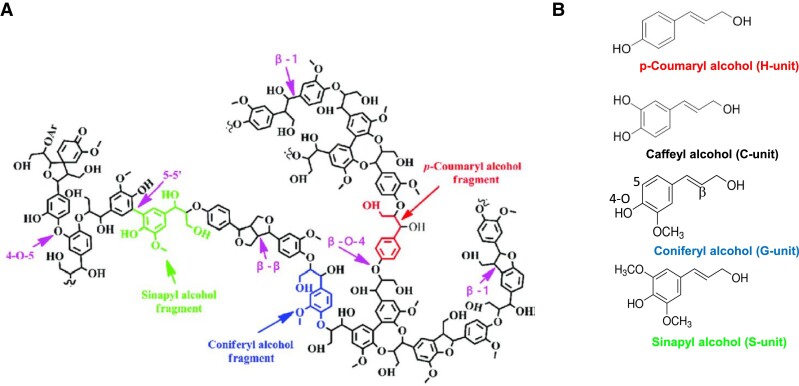
Structure, monomer composition, and linkages of lignin. **A)** A generic lignin molecule showing the major monomer types and the varieties of linkage type. β-*O*-4 linkages are the most common and the easiest to break. Carbon-carbon linkages such as 5-5´ are hard to break and contribute to the recalcitrance of lignin toward degradation. Reproduced from Zakzeski et al. (2010). **B)** The 4 naturally occurring monolignols.

Lignin is a major structural component of plant SCWs. As mentioned above, the cell walls Robert Hooke first described in the 1660s were from cork, and their shape was immortalized by the recalcitrant layers of what was later shown to be suberin, a complex polymer of polyunsaturated fatty acids esterified to phenolic units that is likely anchored to lignin. Suberin makes up 40% of the cork cell wall and lignin comprises another 20%. The term lignin was coined by the Swiss taxonomist Augustin Pyramus de Candolle in 1813. He had isolated a fibrous plant material that was insoluble in water but soluble in alkaline solutions and named it lignin based on the Latin name for wood (lignum). The underlying chemical structure of lignin as a biopolymer of coniferyl alcohol units joined through ether linkages was proposed by Peter Klason in 1897, and the residue after total acid hydrolysis of the carbohydrate components of wood is still termed Klason lignin. In a series of seminal papers between 1940 and 1970, Freudenberg and colleagues established, through chemical analysis and precursor labeling experiments, that lignin was formed primarily from hydroxyphenyl, coniferyl, and sinapyl alcohols (Fig. 11) derived from the amino acid L-phenylalanine linked through free radical coupling (dehydrogenation) (Freudenberg 1959; Freudenberg and Neish 1968). Studies in spruce and magnolia showed that gymnosperm lignins are composed almost exclusively of guaiacyl (G) units, whereas angiosperm lignins are primarily derived from G and syringyl (S) units, with a much lower proportion of hydroxyphenyl (H) units. As a result of their assembly by non-enzymatic polymerization, there is no exact structure for lignin chains. Earlier models suggested very large 3-dimensional structures with a high degree of cross-linking (Freudenberg and Neish 1968), whereas later models favor shorter, more linear, less branched chains (Ralph et al. 2019). The extent of cross-linking will depend on the S/G ratio based on chemical coupling preferences that can be illustrated with in vitro monolignol polymerization systems (van Parijs et al. 2010).

**Figure 12.**
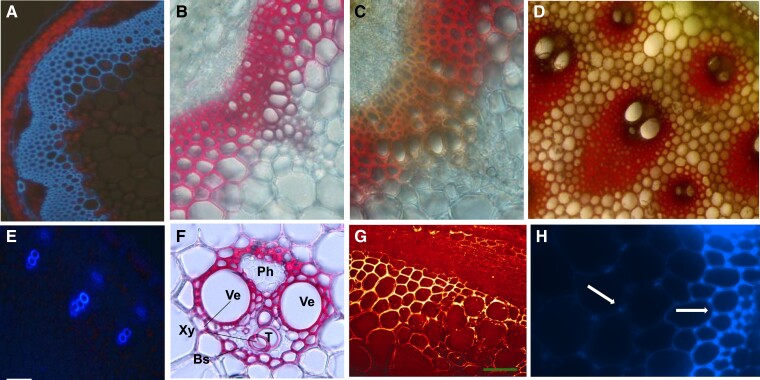
Lignin detection in situ. **A)** Cross section of an Arabidopsis stem viewed under UV light. Lignin autofluorescence is blue, chlorophyll autofluorescence red. **B)** Higher magnification of a cross-section of an Arabidopsis stem stained with phloroglucinol-HCl. **C)** As in **B)** but stained with Maule-reagent. The S-lignin–rich interfascicular fibers stain red, whereas the G-lignin–rich vessel elements stain yellow-brown. A to C are from Wang et al. (2010). **D)** Phloroglucinol staining of a cross-section of a transgenic maize stem with ectopic lignification, showing the different organization of fibers compared with the dicot Arabidopsis. **E)** Cross section of a switchgrass stem viewed under UV light. **F)** Higher magnification of the vascular tissue of a switchgrass stem stained with phloroglucinol-HCl. Bs, bundle sheath cell; Ph, phloem; Ve, vascular element; xy, xylem. D to F are from Gallego-Giraldo et al. (2015). **G)** Forward coherent anti-Stokes Raman scattering (F-CARS) microscopy of a cross section through the epidermal tissue, cortex, and inter-fascicular fiber region of a wild-type alfalfa (*Medicago sativa*) stem. The highest CARS signal is in vascular tissue and can be quantified. From Zeng et al. (2010). **H)** Cross-section through the stem of an alfalfa line undergoing ectopic lignification as a result of downregulation of a WRKY transcription factor. Arrows show lignification in the cell corners of lignified and lignifying cells (L. Gallego-Giraldo and R. A. Dixon, unpublished results).

The linkages between the H, G, and S monolignols can be determined by analysis of partial degradation products or, more conveniently, by NMR (Marita et al. 1999). The most common linkage is the β-*O*-4 linkage between the free 4-OH group of the H, G, or S units and the side chain C2 position of another unit (Fig. 11A), and cleavage at this linkage is the basis for the determination of lignin content and composition by the commonly used technique of thioacidolysis (Lapierre et al. 1985). The several other linkages in lignin are not cleaved by this method. This highlights the problems in the quantitative measurement of lignin; the methods with the most chemical precision are less able to sample the whole molecule, whereas those that rely on simple gravimetric or UV/VIS spectroscopic measurement are prone to interference from contaminants. Conveniently, lignin shows blue autofluorescence under UV light, although similar fluorescence is shown by wall-bound hydroxycinnamic acids. Lignin localization in plant tissues can also be determined by Weisner (phloroglucinol) or Maule (permanganate) staining, the latter showing some specificity for S-lignin, although neither is fully specific or quantitative (Lewis and Yamamoto 1990). Raman spectroscopy provides a means to both visualize and quantify lignin under the microscope (Agarwal and Ralph 1997; Zeng et al. 2010). These various approaches to lignin visualization, along with examples of lignification patterns in stems of both monocots and dicots, are shown in Fig. 12.

It is perhaps surprising that more is not known of the interactions between lignin and the other cell wall components. The current picture is informed by both direct measurements and inferences from indirect approaches such as genetic modification of lignin or other cell wall polysaccharides. It has been generally assumed that lignin assembles on hemicelluloses, and sequential subcritical water extraction of hemicelluloses from birchwood revealed the presence of specific xylan domains that appeared to interact with lignin (Martínez-Abad et al. 2018). This has been confirmed by solid-state NMR measurements of maize stems, where the lignin was shown to be present in hydrophobic nanodomains with electrostatic interactions with the polar motifs of xylans that are not strongly associated with cellulose (Kang et al. 2019).

Sequential glycome profiling of cell wall residues of stems of transgenic plants with altered lignin content and/or composition has shown that both rhamnogalacturonan and xylan epitopes become more water-soluble upon reducing lignin content (Gallego-Giraldo et al. 2018, 2020). Furthermore, biomass from stems of Arabidopsis with reduced lignin content becomes an effective substrate for growth of strains of the thermophilic bacterium *Caldicellulosiruptor bescii* lacking the pectinase gene cluster that is normally required for growth on plant biomass (Gallego-Giraldo et al. 2020). These studies suggest that either lignin is directly associated with pectins and xylan, thereby protecting their accessibility to mild solvent, or that lignin modification brings about active cell wall remodeling leading to greater solubility of other wall polymers, as further discussed below.

It is generally accepted that lignin is found in all higher plants but not in the mosses. Lignin has been identified in at least 1 red alga, where it has been ascribed a role in strengthening cell walls in the genicular tissue linking articulated fronds (Martone et al. 2009). Through studies on delignification of fibers, it has been shown that lignin contributes to tensile strength but has limited impact on bending strength (Wang et al. 2017). Other than during the early stages of lignin deposition in the middle lamella, only plant SCWs are lignified, and such walls are found in xylem, interfascicular fibers, Casparian strip in roots, and some seed coats. Lignin is also found in various other cell types in smaller amounts, including in the walls of anthers and in cotton fibers (Gao et al. 2019). The cell biology of lignification has been well reviewed elsewhere (Barros et al. 2015). Based on the location of lignified walls and more recent studies demonstrating the impairments resulting from genetically engineered reductions in lignin content, the primary functions of lignin are presumed to be the imparting of physical strength, allowing plants to maintain an upright habit or to permit dehiscence of fruits and seeds; preserving a hydrophobic environment to allow passage of water in vessels; and sealing of damaged tissues to prevent pathogen ingress.

The chemical recalcitrance of lignin associated with these functions has been an impediment to the utilization of lignocellulosic materials for conversion of the cellulosic and hemicellulosic cell wall components to liquid biofuels (Li et al. 2016a, 2016b, 2016c; De Meester et al. 2022) and for the digestibility of forage crops (Barros-Rios et al. 2018). At the same time, the realization that lignin could replace petroleum products as a feedstock for the synthesis of biomaterials, high value chemicals, and fuels has led to the concept of lignin valorization (Ragauskas et al. 2014; Martins et al. 2022; Ullah et al. 2022). Together, these concepts have inspired great effort to better understand lignin biosynthesis and its regulation over the past 20 years, which has been the subject of a number of review articles (Dixon and Barros 2019; Ohtani and Demura 2019; Ralph et al. 2019; Vanholme et al. 2019; De Meester et al. 2022).

#### Biosynthesis of lignin

**Figure 13.**
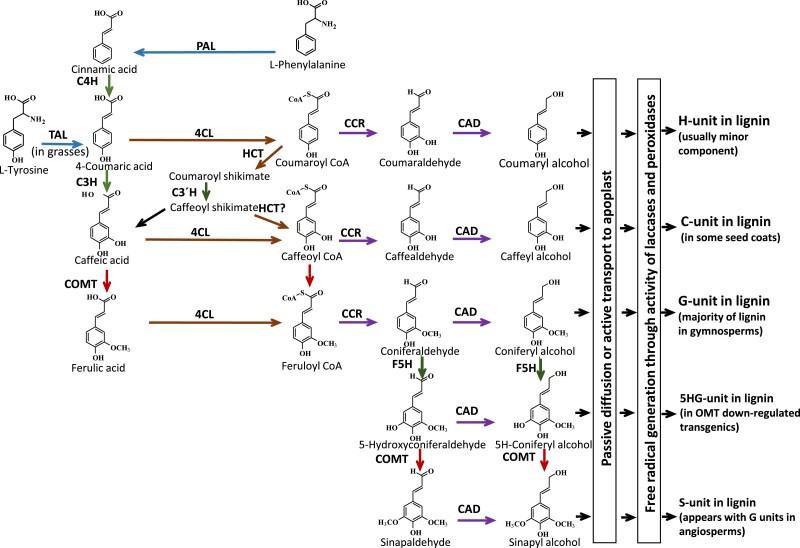
Biosynthetic origins of lignin building blocks, showing enzymatic reactions involved in lignin biosynthesis from primary metabolism (phenylalanine and tyrosine). Arrows are color-coded to show different classes of reactions: blue, deamination; green, hydroxylation; red, *O*-methylation; brown, CoA activation; purple, reduction. Enzymes are PAL, L-phenylalanine ammonia-lyase; TAL, L-tyrosine ammonia-lyase; C4H, cinnamic acid 4-hydroxylase; 4CL, (hydroxy)cinnamate CoA ligase; HCT, hydroxycinnamoyl CoA: shikimate hydroxycinnamoyl transferase (HCT); C3´H, coumaroyl shikimate 3´-hydroxylase; CSE, caffeoyl shikimate esterase, C3H, coumarate 3-hydroxylase; COMT, caffeic acid/5-hydroxyconiferaldehyde 3/5-*O*-methyltransferase; CCoAOMT, caffeoyl CoA 3-*O*-methyltransferase; CAD, cinnamoyl CoA reductase (CCR); CAD, cinnamyl alcohol dehydrogenase; F5H, ferulic acid/coniferaldehyde 5-hydroxylase.

Early models of lignin biosynthesis envisaged a metabolic grid encompassing multiple routes from the first committed precursor, *trans*-cinnamic acid, to the H, G, and S monolignols. Subsequent models, based primarily on the substrate specificities of recombinant pathway enzymes, suggested a more defined set of routes through the grid (Humphreys et al. 1999), but the complexity and flexibility of the pathway has become more apparent recently through new genetic evidence and the application of kinetic and metabolic flux analysis (Vanholme et al. 2013; Wang et al. 2018; Barros et al. 2022). It is now clear that, in grasses, a significant proportion of the flux into lignin originates from tyrosine rather than phenylalanine (Barros et al. 2016). Figure 13 illustrates the currently accepted routes to the monolignols. The genes encoding all the enzymatic steps have been isolated and most of their functions validated through genetic approaches (Dixon and Barros 2019; Vanholme et al. 2019).

Unlike the other cell wall polymers, lignin shows a degree of flexibility as to its monomeric constituents. This first became apparent from the discovery of noncanonical units such the flavanol tricin, stilbenes, or feruloyl putresceine in the lignin from some species (Ralph et al. 2019), stimulating attempts to introduce additional units with more labile bonds to allow for easier cell wall processing. This was achieved via the engineering of pathways for the formation of artificial monolignol ferulate and the diferuloyl methane curcumin, which were transported to the apoplast and incorporated into lignin, resulting in enhanced saccharification of biomass (Wilkerson et al. 2014; Oyarce et al. 2019). Incorporation of a pathway for formation of *p*-hydroxybenzoate (*p*HBA) resulted in a large increase in esterified *p*HBA in poplar lignin, with potential as a “clip-off” molecule for processing to downstream high value chemicals (Mottiar et al. 2023).

Lignin is polymerized in the apoplast after the pectic, hemicellulosic, and cellulosic components have been laid down following their transport through the Golgi system (pectin and hemicellulose) or synthesis by a trans-membrane molecular “machine” (cellulose). This raises a number of questions concerning the transport of lignin monomers and the initiation of their polymerization. Based on the presence of high levels of coniferin (coniferyl alcohol β-D-glucoside) in xylem sap and its temporal association with lignification in the developing xylem of coniferous trees, along with results of radiolabeling experiments (Terashima and Fukushima 1988), it was proposed that monolignols were transported to the apoplast as their glycosides utilizing membrane transporters and then underwent conversion back to the aglycones through the activity of an apoplastic glycosidase (Tsuyama and Takabe 2015). Biochemical studies suggested the presence of ATP-dependent transporters for coniferin and other monolignol glycosides in membrane preparations from a number of woody species as well as Arabidopsis (Miao and Liu 2010; Tsuyama et al. 2013), but, to date, the only transporter characterized at the molecular level is a low-affinity H-monomer transporter (Alejandro et al. 2012). There is still no genetic evidence for the existence of G- or S-unit transporters, and an alternative hypothesis of passive diffusion down a concentration gradient has been proposed, supported by theoretical thermodynamic models (Vermaas et al. 2019) and by the observation that polymerization to lignin appears to be required for movement of monolignols out of the cytosol to the apoplast (Perkins et al. 2019, 2022; Zhuo et al. 2022). Glycosylation of small monolignol oligomers appears to be required for their intracellular transport from cytosol to vacuole (Dima et al. 2015). A passive diffusion mechanism, or highly promiscuous transporters, are consistent with the ability of “unnatural” monolignols to pass to the apoplast and be incorporated into lignin (Wilkerson et al. 2014; Ralph et al. 2019). The “good neighbor” hypothesis suggests that monolignols may be generated in parenchyma cells adjacent to lignifying xylem elements, then diffusing into and through the apoplast, where lignification is initiated at the cell corners (Fig. 12H) and/or in the middle lamella. In contrast, lignification in interfascicular fibers appears to be cell autonomous (Smith et al. 2013).

The polymerization of monolignols was at first thought to be initiated by the activity of cell wall peroxidases (Freudenberg and Neish 1968), consistent with the cellular localization of these enzymes and the well-studied formation of lignin “dehydrogenation polymers” generated in vitro from monolignols, peroxidase and hydrogen peroxide (e.g. van Parijs et al. 2010). Because the peroxidases are encoded by large gene families (73 members in Arabidopsis) with potential redundancy, it has been difficult to ascribe functions by genetic experiments. It was later proposed that another group of copper oxidases, the laccases, were also likely involved in monolignol polymerization (Sterjiades et al. 1992; Bao et al. 1993). This suggestion has received considerable support from genetic loss of function experiments. For example, lignification is completely absent in stems of the Arabidopsis *lac4 lac11 lac17* triple mutant (Zhao et al. 2013). Genetic analysis has, however, now confirmed roles in lignification for some peroxidases (e.g. Zhang et al. 2022a), and the current model proposes involvement of both enzymes in most lignin biosynthesis, with an exception in the Casparian strip of the root, where peroxidase alone is believed to be necessary (Lee et al. 2013).

Assuming passive transport of monolignols to the apoplast, the localization of the enzymes that initiate free radical formation for polymerization could be critical for determining the site of initiation of lignification. Lignification often starts in the cell corners (Turlapati et al. 2011), and Arabidopsis PEROXIDASE 64 localizes to this region, whereas LACCASE 4 is immobile and localized to the thick secondary wall (Chou et al. 2018). In view of the lack of lignin in the *lac* triple mutant, this suggests that simple initiation of polymerization in the cell corners is insufficient to prime growing lignin chains throughout the wall and that other initiation sites are likely present. Understanding how lignification is initiated in different cell types is a critical question for the future. The pioneering work of Niko Geldner's laboratory has defined the Casparian strip as model for understanding the cell biology and biochemistry of lignin initiation (Barbosa et al. 2019).

Unlike most other plant polymers, lignin is assembled non-enzymatically. The potential lack of any enzymatic control over such an important process led some experts in the field to suggest that the free-radical-based chain elongation might require some kind of template to allow for correct assembly (Davin and Lewis 2000). This view was inspired by the seminal finding of Norman Lewis’ group that oxidative coupling of 2 coniferyl alcohol units to form dimeric lignans was under strict stereochemical control in most plants and that this control was engendered by the co-action of a so-called dirigent protein with the laccase that generated the monolignol radicals (Davin et al. 1997). Dirigent proteins are trimeric proteins with no identified catalytic reaction, but which direct the stereochemistry of radical-based coupling (Davin et al. 1997). They have been found in nearly all plant species, localize to the cell wall, and are often associated with disease resistance (Paniagua et al. 2017). For several years, a heated debate took place as to whether monolignol assembly for lignin (as opposed to lignans) was under chemical control only, as promoted by John Ralph, or required some kind of template that was composed of an array of dirigent sites (supported by Norman Lewis, Simo Sarkannen, and others). The arguments on both sides can be found in Ralph et al. (2008). Although the pervading view now is that lignin chain assembly is essentially purely chemical (and lignin clearly does not possess any recognizable stereochemistry), the idea of a function for dirigent proteins in some aspects of lignification (initiation or localization) is gaining support. For example, maize mutants lacking a specific dirigent protein displayed impaired deposition of lignin in the Casparian strip, leading to reduced salt tolerance (Wang et al. 2022a, 2022b).

The template hypothesis also posited that the H, G, and S monolignols were the only components that could be assembled into “true” lignin, consistent with steric limitations for a template-driven process. The recent discovery that a wide variety of chemically enabled components (tricin, stilbenes, coniferyl, ferulate, curcumin, etc.) can be incorporated into lignin appears to undermine this tenet of the template hypothesis but still leaves open the possibility of specific proteinaceous initiation sites for lignin chain growth. The flavonol tricin has been proposed to serve as an initiation site for lignification in the grasses, although the effects of manipulating its levels through genetic means differ depending on species (Lam et al. 2021). Nevertheless, the flexibility of lignin structure provides an unprecedented opportunity to fine-tune the composition of a major cell wall structural polymer to either assist biomass degradation or promote its valorization.

## Multiple structures involved in many functions

### Models of cell wall organization

Studies of cell wall architecture by Roberts and McCann in the early 1990s produced a series of fast-freeze, deep etch, rotary-shadowing images of onion parenchymatous cell walls (McCann et al 1990) that greatly influenced thinking about how the cell wall is arranged. Such images revealed multi-lamellate 100-nm-thick primary walls with 10-nm pores and cellulose MFs (possibly with associated hemicellulose) of 5- to 12-nm diameter. Importantly, the measured lengths of the isolated polysaccharides were in the 700 nm range, enough to span the wall and enable cell to cell wall cross-links. Already it was recognized that the “spaghetti type” models of the primary cell wall were problematic. “It is clear that the classical description of cellulose microfibrils embedded in an amorphous matrix (Varner and Lin 1989) is conceptually problematic; microfibrils are cross-linked together; all wall components are long rod-like fibres; only removal of cellulose with cellulase gives an image that could be described as an amorphous matrix. It is the nature of the cross-links that remains to be determined” (McCann et al. 1990, p330).

Any comprehensive model of wall structure must answer the crucial unsolved problem of how the various components found in plant cell walls are organized into a matrix that carries out the diverse functions that walls perform. Despite many efforts, with most models focusing on how growth is accomplished, the current “models” are inadequate. Most are descriptive diagrams that aim to depict important structure-function relationships. To be useful, such models should be sufficiently specific that they allow interpretation of experimental data and allow the formulation of predictions that can be experimentally tested. Only recently have models become sufficiently sophisticated that they allow quantitative explanations and predictions (Zhang et al. 2021c).

Over the years, one of the major uses for cell wall models was to explain the reorganization of wall components during elongation growth, especially auxin stimulated growth (see Du et al. 2020b for a recent review). More than half a century ago, it was demonstrated that elongation could be stimulated in many systems by acidic conditions (Rayle and Cleland 1970). It is now understood that auxin stimulates a plasma membrane–localized proton pump that acidifies the cell wall (Du et al. 2020b). What is still not completely resolved is how low pH reorganizes wall components to allow growth.

One of the first efforts to describe such structure/function relationships was the “multi-net” hypothesis put forward in the early 1950s by Roelofsen and Houwink (1953). Its main emphasis was to explain the changes in orientation of cellulose MFs as plant cells undergo anisotropic elongation. They observed that elementary fibrils near the plasma membrane of cotton hairs, and other cells, were oriented transversely, but as cells elongated, the MFs gradually changed their orientation and were mainly axial in outer layers of the primary walls.

**Figure 14.**
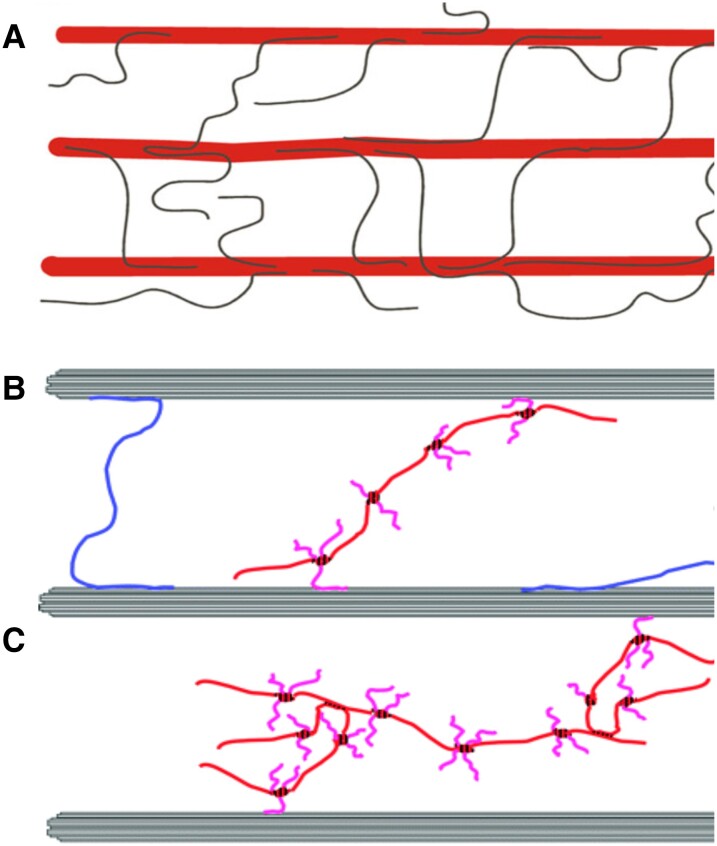
Variations of the tethered network hypothesis using different matrix polysaccharides. **A)** The tethered network hypothesis shows CMFs (thick horizontal rods) to be well spaced and connected by XyG (thin lines). Taken from Cosgrove (2022). **B)** CMFs (thick horizontal rods) linked by both XyG (thin undecorated lines) and pectin (thin line decorated with side chains). **C)** CMFs (thick horizontal rods) linked via a network of pectic polysaccharides (thin lines decorated with side chains). Panels B and C are taken from Zykwinska et al. (2007).

A significant step forward came in the early 1970s with the models emerging from the laboratory of Peter Albersheim, one of the great pioneers of cell wall biochemistry; they incorporated growing knowledge of the structures of wall matrix polysaccharides. One model emphasized the cellulose/XyG/RG-I network as a major source of wall strength that resists the forces of turgor pressure but can be reorganized to allow expansion as cells grow (Albersheim 1975). An early version of this model also included experimentally supported connections of pectic molecules to cell wall proteins (Keegstra et al. 1973). However, the proposed XyG to RG-I connection could not be identified, so the emphasis in subsequent models switched to a cellulose/hemicellulose network (Hayashi 1989). Further refinements were made by others, including describing the differences between Type I and Type II walls (Carpita and Gibeaut 1993), by making the dimensions of the primary wall thickness (50–100 nm) proportional to that of the plasma membrane (7–8 nm) (McCann and Roberts 1991), or by showing the various components in the proper proportions to cellulose content (Somerville et al. 2004). All these models postulate that the strength of the wall is determined by a cellulose/hemicellulose network. Many authors have called this model the tethered network hypothesis (Fig. 14A) (reviewed in Cosgrove 2022). But, as described below, several lines of evidence now argue against its validity (Cosgrove 2014, 2022), so new ideas are needed.

The tethered network hypothesis popularly featured XyG, the most abundant hemicellulose in the primary walls of many plant species (Keegstra et al. 1973; Hayashi 1989; Albersheim et al. 2011; Hayashi and Kaida 2011). In the context of XyG tethers connecting MFs (Fig. 14A), several ideas were considered as to how the network was reorganized during growth. One popular idea was the action of XyG transglucosylase hydrolase (XTH) proteins that cleave or reorganize the XyG tethers (Rose et al. 2002). More recent evidence argues against a role for XTHs in cell expansion, but they may have roles in plant responses to biotic and abiotic stresses (Ishida and Yokoyama 2022). Another option for reorganizing the network came with the breakthrough discovery of expansins, proteins that stimulate cell expansion in a pH-dependent manner without breaking covalent bonds (McQueen-Mason et al. 1992). Expansin proteins are encoded by a large family of genes in plants (Sampedro and Cosgrove 2005). Despite a great deal of molecular information about expansin proteins, the molecular details of how they achieve elongation growth remains unclear (Cosgrove 2015, 2016).

An Arabidopsis double mutant (*xxt1/xxt2*; Cavalier et al. 2008) and a triple mutant (*xxt1/xxt2/xxt5*; Zabotina et al. 2012) with disruptions of genes responsible for adding xylose to the XyG backbone have XyG levels below the limit of detection. But these mutant plants grow and develop with only minor defects, casting doubt on the validity of the hypothesis that XyG plays an important role in defining the mechanical properties of primary cell walls. More recently, an Arabidopsis quintuple mutant with disruptions of all 5 *CSLC* genes, encoding proteins that synthesize the glucan backbone of XyG, has undetectable levels of XyG but can grow and develop with minor altered phenotypes, including altered root hairs and pollen tube growth (Kim et al. 2020).

It is noteworthy that the presence of aberrant forms of XyG is more detrimental to plant growth and development than the lack of XyG. The Arabidopsis *mur3-3* mutant that lacks fucose and has very low levels of galactose on XyG exhibits severe dwarfism and other growth phenotypes (Kong et al. 2015; Xu et al. 2017). But crossing the *mur3-3* mutant to the *xxt1/xxt2* double mutant lacking XyG restores the normal size of the triple mutant plants (Kong et al. 2015). These findings emphasize the importance of the physical properties of wall polysaccharides in determining polymer interactions.

Several groups have utilized the Arabidopsis mutants lacking XyG to investigate cell wall functions related to expansion growth. The walls in *xxt1/xxt2* mutant plants were examined in detail and found to be more extensible than wild-type walls using stress/strain assays but less extensible in creep mediated by α-expansin (Park and Cosgrove 2012b). The same group used multiple different endoglucanases to treat tissues from wild type and *xxt1/xxt2* mutant plants followed by examination of wall biomechanical properties. Based on their observations, they concluded: “Our results are incompatible with the common depiction of xyloglucan as a load-bearing tether spanning the 20- to 40-nm spacing between cellulose microfibrils….” To explain their observations, they postulated that “…XyG-mediated control of wall extension may be restricted to relatively inaccessible contact surfaces between microfibrils of the primary cell wall” (Park and Cosgrove 2012a, p1933. Later publications referred to these contact surfaces as “biomechanical hotspots” (Cosgrove 2022). One version of the hypothesis assumes that residual XyG is present in the walls of the mutant plants, which has not been demonstrated. Another possibility for the “biomechanical hotspots” is that they consist of direct interactions between cellulose MFs as described by Cosgrove (2022). In this case, the role of matrix polysaccharides, whether XyG, other hemicelluloses, or pectins, is to prevent MF from coalescing into bundles that form a rigid inflexible network. A different strategy was used by Kuki et al. (2020), who prepared protoplasts from the leaves of Arabidopsis *xxt1/xxt2* mutant plants. As protoplasts regenerated cell walls, they observed “…only a slight difference in the structure of cellulose microfibril network between *xxt1*/*xxt2* and wild-type (WT) protoplasts.” From these and other observations, they concluded that “…xyloglucan is not essential for the initial assembly of the cellulose network, and the cellulose network formed in the absence of xyloglucan provides sufficient tensile strength to the primary cell wall regenerated from protoplasts.” (Kuki et al. 2020, p. 1)

One possible explanation of the ability of plants to survive the lack of XyG is that other wall molecules can substitute for it, that is, functional redundancy. Sowinski et al. (2022) explored this possibility by performing a detailed analysis of the composition of the *xxt1/xxt2* double and *xxt1/xxt2/xxt5* triple mutants. They observed a small increase in the levels of glucomannan, but significant increases in the levels of pectic polysaccharides. Interestingly, these increases were not caused by any major increases in transcripts responsible for biosynthesis of these polymers. Yu et al (2022) have suggested that a specific galactoglucomannan may function like XyG. Mutants lacking the ability to make this mannan are nearly normal, but when crossed with the *xxt1/xxt2* mutant, the triple mutant has more severe phenotypes than mutants lacking either polymer (Yu et al. 2022) but is still able to grow and complete its life cycle.

Despite the evidence against the tethered network involving cellulose and XyG, there are several reasons why it has proven difficult to abandon this model. First, the structure of XyG is highly conserved in all land plants, arguing that it has an important conserved function. Second, XyG is abundant in plants with Type I PCWs, although its abundance varies greatly from one species to another and sometimes within the tissues of a single species, and its abundance is low in plants with Type II PCWs. Yet despite the abundance of XyG and its conserved structure, no one (including the authors) have yet proposed a plausible hypothesis for its role in PCWs.

Another version of the tethered network hypothesis proposes that pectic polysaccharides either share duties with XyG (Fig. 14B) or replace it (Fig. 14C) in forming tethers between cellulose MFs (Zykwinska et al. 2007). Although this hypothesis has not received the same level of attention, we argue that this hypothesis, or some variation of it, deserves attention considering the phenotypes of the XyG-deficient mutants.

During his thesis work in the laboratory of James Bonner at Caltech, Peter Albersheim explored the possibility that auxin enhances cell elongation by changes in the metabolism of pectins (Albersheim and Bonner 1959; Jansen et al. 1960). They focused on changes in the extent that galacturonic acid is methyl esterified during auxin treatment of oat coleoptiles. Although primitive by today's standards, this early work examined some of the same ideas for how pectins might regulate cell expansion that are being pursued today.

Boyer and colleagues studied the growing internodes of *Chara corallina,* a green alga with a higher plant-like cell wall (Domozych et al. 2010). In a series of experiments spanning 13 years, they provided compelling evidence that in *Chara* calcium pectate wall polymers provide the critical interactions that regulate cell expansion (Proseus et al. 1999, 2000; Proseus and Boyer 2005, 2007, 2012a, 2012b). They proposed a “calcium pectate cycle” model describing calcium-pectate (HG) as the load-bearing component in the *Chara* wall.

In their review of cell wall mechanics and plant cell growth, Peaucelle et al. (2012) made the compelling argument that the mechanism of cell wall extensibility in Charophycean algae, that is, the coupling of cell wall synthesis to cell wall extensibility through calcium-exchange between existing and newly synthesized calcium-pectate, also exists in terrestrial plants and called that mechanism the “ancient process.” They further proposed that land plants have at least 2 load-bearing constituents, a calcium-pectate component, and a cellulose-xyloglucan network and that the latter is associated with an expansin-dependent process. They proposed that the more recent expansin-based process “amplifies” the calcium-pectate mechanism and “allows faster growth.”

These ideas were explored in a series of studies examining the elongation of dark-grown Arabidopsis hypocotyls (Pelletier et al. 2010; Peaucelle et al. 2015; Daher et al. 2018). In one study, AFM was used along with antibodies that distinguish ME-HG from de-esterified HG (DE-HG) in plants where expression of PME and PMEIs had been manipulated. Although space constraints preclude a detailed explanation of the study, the authors concluded that “…growth symmetry breaking is controlled at a cellular scale by bipolar pectin de-methylesterification…” (Peaucelle et al. 2015, p. 1746. In a follow-up study using the same experimental system, Daher et al. (2018) also found asymmetry in wall elasticity in cells that was coincident with growth anisotropy and correlated with changes in pectin HG de-methylesterification. They concluded that pectin asymmetry in the hypocotyl epidermis contributes to anisotropic growth.

Jönsson et al. (2021) studied the bending of the apical hook after emergence of the hypocotyl. This process is known to be regulated by auxin and involves differential growth on the 2 sides of the hypocotyl. Again, space constraints preclude a complete description of the experiments, but the authors found a “…spatial correlation between asymmetric auxin distribution, methyl esterified HG pectin, and mechanical properties of the epidermal layer of the hypocotyl in Arabidopsis.” (Jönsson et al. 2021, p. 1154)

In a ground-breaking study of expansion in Arabidopsis epidermal pavement cells, Haas et al. (2020) used super-resolution 3-dimensional direct stochastic optical reconstruction microscopy, cryo-scanning electron microscopy, and HG-specific antibodies that distinguish ME-HG versus DE-HG to show that in cotyledon anticlinal walls HG is organized as HG nanofilaments proposed to be similar to the pectate multichain helical crystalline fibers described by Walkinshaw and Arnott (1981a, 1981b). They further showed that the dimensions of DE-HG nanofilaments are 1.4 times greater than the ME-HG nanofilaments and proposed that de-methylesterification of HG nanofilament quaternary structure leads to pavement cell expansion. They call this the “expanding beam” model of pavement cell expansion. They further tested this model through development of a 3D nonlinear finite element method model, which predicted the pavement cell growth observed. Further tests of the model via in silico variation in level of methyl esters on HG by overexpression of PME and PMEI showed that, as expected, overexpression of PMEI led to growth inhibition.

More recently, Haas et al. (2021) hypothesized that enzymatic de-methylesterification of HG to induce calcium-crosslinked HG in the cell wall, in addition to turgor pressure, drives phase separation in cell wall architecture and associated plant cell expansion. Depending on the methylesterification state, HG is either highly partially or relatively poorly charged. As HG appears to be highly methylesterified in vivo when it is synthesized and inserted into the wall, its de-esterification by wall-located PMEs can lead to increases in charge and, when calcium is present, salt bridges between calcium and adjacent HG chains. This HG de-methylesterification leads to associated changes in HG quaternary structure and volume transition (Haas et al. 2020) akin to phase separation.

Based on the evidence summarized above and explained in more detail in various reviews (Peaucelle et al. 2012; Daher and Braybrook 2015; Braybrook and Jönsson 2016; Chebli and Geitmann 2017; Hocq et al. 2017a; Haas et al. 2021; Höfte 2023), pectins play an important role in defining cell shape and regulating growth. In addition, increasing amounts of NMR data provide evidence for abundant interactions between cellulose and pectic polysaccharides (Pérez García 2011; Wang et al. 2015; Wang and Hong 2016; Phyo et al. 2019; Temple et al. 2022). Furthermore, genetic evidence supports an important role for pectins. In contrast to the various mutants lacking XyG that have only minor phenotypes, several pectin-deficient mutants have severe phenotypes (Bouton et al. 2002; Mouille et al. 2007; Du et al. 2020a) and others may be lethal (Caffall et al. 2009). The important conclusion is that pectic polysaccharides must be included in models that attempt to explain how wall structure is reorganized during growth. More detailed knowledge about pectin structure as it exists in PCWs is critical to understanding cell growth.

Current efforts to generate a model of the PCW have similarities to the parable of a group of blind people trying to describe an elephant with each person describing their part: a leg, a trunk, a tusk, etc. The problem is made worse by the recognition that PCWs are dynamic structures with many different functions and stages in their life, as described in the following sections.

### Cell wall signaling

Perhaps the biggest surprise over the past few decades has been the realization that the cell wall is not the passive structure we assumed but is actively engaged in signaling processes. Plants are constantly under changing stresses, be they abiotic (e.g. salinity, drought, tensile) or biotic. Because degradation of plant cell walls is often a necessity for establishing infection, it seems intuitive that the cell wall would possess sensing mechanisms for cell wall integrity (CWI) that could recognize damage associated with either pathogen ingress or wounding. Even the act of cell elongation involves processes of wall loosening that may create tensile stress (Wolf 2022). Historically, the field of cell wall signaling developed from 3 distinct approaches: the identification of mobile signals within the cell wall such as cell wall–derived oligosaccharide elicitors of plant defense responses (e.g. Ayers et al. 1976); the manipulation of the level of specific wall polymer types that trigger compensatory changes in wall composition (e.g. Hematy et al. 2007); and genetic screens for genes underlying developmental phenotypes, which revealed several key plasma membrane signaling receptors (e.g. Boisson-Dernier et al. 2009; Guo et al. 2009, Duan et al. 2010).

Communication between the extracellular matrix and the cytosol is largely brought about through the binding of ligands that activate PM-localized receptor-like kinases (RLKs), with their ligand-binding domains located within the wall matrix (Wolf 2022). Plants contain large numbers of such transmembrane receptors, many of which, such as the LRR-receptor kinase resistance (R) genes or wall-associated kinases (WAKs), are associated with the recognition of pathogens (McHale et al. 2006; Cho et al. 2022). Other mechano-sensitive receptors sense physical features of the PM-wall continuum such as turgor or tensile stress (Hamant et al. 2019; Bacete and Hamann 2020), whereas others, such as the class referred to as CrRLK1 receptors, physically interact with cell wall components such as pectin or peptides (Bacete et al. 2018; Zhu et al 2021). Remodeling of the cell wall is the process that closes out the cycle of cell wall signaling. The involvement of jasmonic acid (JA) and salicylic acid (SA), and their cross-talk with other hormones such as ABA and ethylene in control of cell wall remodeling, is well-reviewed elsewhere (Vlot et al. 2009; Tenhaken 2014; Shigenaga et al. 2017; Ali and Baek 2020; Anderson and Kieber 2020; Liu and Timko 2021).

With no intention of covering this complex field in depth, we focus primarily on initial events that occur within the cell wall and the cell wall-PM interface. We discuss causes of loss of CWI, examples of the roles of tensile stresses in growth/CWI signaling, elicitors and some key receptors and co-receptors, and end with examples of signaling in SCWs. A number of recent reviews cover the broader field in more detail (Bacete et al. 2018; Rui and Dinneny 2019; Vaahtera et al. 2019; Anderson and Kieber 2020; Bacete and Hamann 2020; Wolf 2022; Colin et al. 2023). Unless indicated otherwise, studies were carried out in Arabidopsis and focused on PCWs. Because the field is moving so fast, it is almost guaranteed that, by the time this review appears, there will have been exciting new breakthroughs.

#### Causes of loss of CWI

One of the most dramatic early examples of the amazing plasticity of cell walls in response to stress is that of Shedletzky et al. (1990, 1992) who showed that cultured cells of tomato, tobacco, or the grass Lolium can slowly adapt and compensate for the loss of cellulose caused by growth on the cellulose synthesis inhibitor DCB. The dicot cells compensated by creating walls comprised almost exclusively of pectin and proteins, while the grass, lacking much pectin, largely enhanced phenolic cross-linking of arabinoxylan. Similar studies with cultured Arabidopsis cells using ISX [that inhibits CSC activity but not that of the CSLD proteins that also synthesize cellulose (Yang et al. 2020b; Wu et al. 2023)], showed less dramatic decreases in cellulose, similar increases in pectin, but also upregulation of CSLD5 and a collagen-like, gly-rich protein (Manfield et al. 2004).

Because cellulose is a major load-bearing polymer in PCWs, it is not surprising that short-term inhibition of its synthesis also has notable consequences (Wang et al. 2016a; Kesten et al. 2017; Wolf 2022). These include swelling of expanding cells, inhibition of elongation, changes in levels and composition of pectins, and sometimes but not always, deposition of callose or ectopic lignin. Natural causes of cellulose synthesis inhibition might include tensile stresses that affect mechanosensitive calcium channels (MCAs), disruption of CSC-MT associations, salt/osmotic stress, or many poorly understood conditions relating to the extreme lability of rosette CSCs. Plants can also detect stress-induced alterations in pectin content or structures due to the presence of heavy metals (Muschitz et al. 2015) or high salinity, which can trigger de-methylesterification of loosely bound pectins, MT degradation, and loss of cellulose (Gigli-Bisceglia et al. 2022; Colin et al. 2023). The middle lamella is rich in DE-HG, and mutations in *qua1*, a GAUT, and *qua2*, a PMT, can affect cellulose synthesis and other phenotypes due to increased cell separation (Du et al. 2020a). Cell wall–degrading enzymes from pathogens play a key role in releasing small signaling molecules and altering CWI, especially through attack on cellulose and pectins. There is less evidence for sensing in response to altered levels of PCW XyG or structural proteins, although LRXs and Gly-rich proteins have been implicated in signaling (Park et al. 2001; Manfield et al., 2004; Herger et al. 2019). As described later, engineered changes to lignin in SCWs can surprisingly also lead to large effects.

#### Oligosaccharin elicitors as mobile signal molecules

Studies on defense signaling began with the pioneering work of Peter Albersheim and his students and post-docs on elicitor-active oligosaccharides from plants and pathogenic fungi (reviewed by Côté and Hahn 1994). Not particularly welcomed coming from a young intruder from the cell wall world into the phytopathology community, the Albersheim hypothesis has nevertheless proven largely valid. This work developed from the elicitor hypothesis for induction of plant defense responses by fungal cell wall components but was extended to include plant developmental responses (Augur et al. 1992) and evidence that pectin-derived fragments signal loss of CWI beyond that caused by pathogens (Wolf 2022). Elicitor-active cell wall–derived oligosaccharides include hepta-glucosides from the cell walls of *Phytophthora sojae*, a range of oligogalacturonides (OGAs) from various sources, xyloglucan oligosaccharides (Ayers et al. 1976; Davis et al. 1986; Fry 1986, 1994), and even a grass-specific MLG-derived elicitor (Yang et al. 2021). Depending on how they are generated, such elicitor molecules are now generally referred to as Damage Associated Molecular Patterns or Pathogen Associated Molecular Patterns (Boutrot and Zipfel 2017; Bacete et al. 2018). The early studies on oligosaccharins were essentially pharmacological, and, although providing reagents for studying defense gene induction, did not provide understanding of their biological significance or mechanism of action. Furthermore, methods for accurately determining the complete repertoire of wall-released molecules were in their infancy. Recent improvements in liquid chromatography-mass spectrometry are now making it easier to find in vivo players and link them to their in vivo bioactivities (Voxeur et al. 2019; Liu et al. 2023).

Recent work on oligosaccharins showed that a family of enzymes called lytic monosaccharide oxygenases (LPMOs) found in pathogens can cleave recalcitrant and crystalline polysaccharides such as cellulose through oxidative attack. One group of LPMOs (AA9) was shown to produce oxidized cellobiose and larger oxidized cellodextrins, and a recent study demonstrated that plants perceive the unoxidized and oxidized forms through different signaling pathways and that a combination of both cellobiose and oxidized cellobiose provided better resistance to necrotrophic infection than either alone (Zarattini et al. 2021).

#### Peptide elicitors

One major class of peptide elicitors that has been recently recognized as central to cell wall signaling is a family of peptides called RALFs. The first of these was discovered accidentally by Clarence “Bud” Ryan during isolation of the small systemin peptide that induces wound responses in Solanaceous plants; during isolation, a different, larger 5-KDa peptide was also discovered that caused even more rapid alkalinization of the apoplast than systemin (Pearce et al. 2001). RALF peptides are now known to have diverse signaling roles related to alteration of apoplastic pH and growth and/or defense (Haruta et al. 2014; Xiao et al. 2019; Blackburn et al. 2020). Some RALF peptides are generated and activated by proteolysis of propeptides, whereas others have no propeptide sequence or cleavage site, characteristics that can affect the nature of their signaling activity (Stegmann et al. 2017). Another small family of peptides in Arabidopsis referred to as Peps also plays a key role in immune and other types of signaling (Bartels and Boller 2015; Dressano et al. 2020) leading to classic responses such as elevation of intracellular Ca^2+^, ROS, and production of defense-related hormones such as JA and SA. In a positive feedback loop, they are generated from propeptides by Ca^2+^-dependent metacaspases (Shen et al. 2019).

#### Plasma membrane receptors

To understand the receptors for CWI signaling that have been discovered in recent years, we first need to meet a cast of classical Greek and Etruscan characters, starting with FERONIA (FER), named after the Etruscan goddess of fertility, and others to be discussed later such as THESEUS1 (THE1), ERULUS (ERU), and HERCULES (HERK). These are members of a clade of 16 PM-localized proteins referred to as CrRLK1Ls that are within the superfamily of RLKs (Cheung and Wu 2011; Nissen et al. 2016; Zhu et al. 2021). CrRLK1L PM-localized receptors are characterized by their extracellular motifs resembling sugar-binding domains called malectins coupled to an intracellular kinase domain (Nissen et al. 2016). Depending on what ligands are bound, the receptors often interact through phosphorylation of guanine nucleotide exchange factors (GEFs) that enhance the downstream activity of plant-specific small GTPases called ROPs (Berken 2006). Further ROP signaling can lead to many other possible downstream events such as activation or inhibition (depending on the CrRL1K/GEF/ROP combination) of NADPH oxidase to produce ROS and of Ca^2+^ channels that when activated elevate [Ca^2+^_cyt_] that can promote exocytosis of wall precursors and activate other downstream signals that lead to production of JA or SA (Chen et al. 2020a, 2020b; Tang et al. 2022). In turn, these hormones can lead to upregulated expression of proteins involved in defense responses or cell wall remodeling (Vlot et al. 2009; Ali and Baek 2020; Liu and Timko 2021).

##### FERONIA—a scaffold on which to create diverse signaling pathways

**Figure 15.**
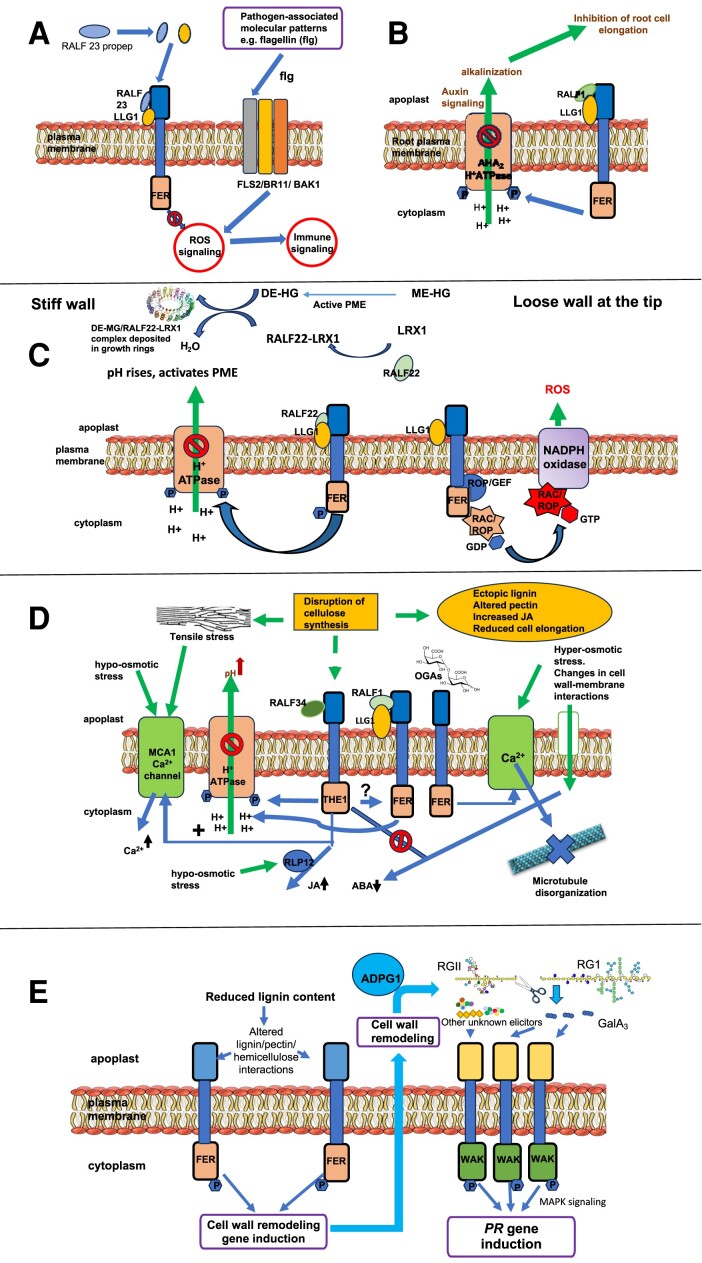
Examples of how variation in ligands and receptor/co-receptor combinations control cell wall signaling outcomes. **A)** A FER-LLG1-RALF23 combination inhibits a PAMP-mediated ROS production and subsequent immunity signaling (Stegmann et al. 2017; Xiao et al. 2019). **B)** The work of Haruta et al. (2014) demonstrated that RALF1 binding to FER complex (that was later shown to include co-receptor LLG1) leads to phosphorylation and inhibition of AHA_2_ PM-ATPase. This leads to alkalinization of the cell wall and inhibition of root cell elongation. Later work indicates other RALF1-binding proteins may also be involved (not shown, see Dressano et al. 2017). **C)** The role of FER and an LRX1-DE-HG complex in regulating root hair elongation. Loss of function of ERULUS also regulates FER and AHA10 phosphorylation (not shown). Relevant references are Duan et al. 2010, Li et al. 2015, Schoenaers et al. 2018 and 2023. **D)** Examples of different roles for ligands and/or mechano-sensing by THE1, FER, and Ca^2+^ channels for responses to CWI and for the complex regulation involving RLP12 of hypo- (through JA) and hyper-osmotic stresses (through ABA). Relevant references are Hematy et al. (2007), Engelsdorf et al. (2018), Feng et al. (2018), Hamant et al. (2019), and Bacete et al. (2022). **E)** Induction of PR proteins as a result of engineered reduction of lignin content. FER senses altered wall integrity to induce the polygalacturonase ADG1, which releases GalA_3_ and other elicitors from pectin; these are recognized by WAKs resulting in induction of PR1 and PR10 (Gallego-Giraldo et al. 2020; Liu et al. 2023).

FER was first discovered in studies of pollen/stigma interactions (Huck et al. 2003). Because it has since been implicated in signaling for a multitude of pathways, FER has been described as a “scaffold” that can interact with different ligands (e.g. RALF peptides, OGAs) and/or co-receptor combinations to control different signaling pathways. The remarkable number of pleiotropic responses signaled by 1 FER protein and its partners are reviewed in depth elsewhere (Cheung and Wu 2011; Li et al. 2016a; Vaahtera et al. 2019; Chen et al. 2020a, 2020b; Xie et al. 2022). FER can play a role in inhibiting immune signaling in several ways. Figure 15A shows 1 example of how certain ligand and protein partners inhibit the outcome of PAMP-mediated ROS production that is signaled through a FLS2/BR11/BAK1 complex. Inhibition occurs when the pro-peptide of RALF23 is cleaved, and the resulting RALF23 interacts with the GPI-anchored co-receptor protein LLG1 (Li et al. 2015) that in turn interacts with FER, and this complex in turn inhibits PAMP-induced ROS production and subsequent immune signaling (Stegmann et al. 2017; Xiao et al. 2019) (Fig. 15A).

Two other examples compare FER's partner combinations for control of the auxin-regulated growth of elongating root cells with the complex and differing configurations used to regulate tip growth of root hairs. A study by Mike Sussman's group showed that when FER-LLG1 associates with RALF1, it leads to suppression of cell elongation through alkalinization of the cell wall through FER phosphorylation-induced inhibition of the PM H^+^-ATPase AHA_2_ (Haruta et al. 2014; Li et al. 2015) (Fig. 15B). Root hair tip growth is a somewhat different process that shows oscillations in which the growth phase is promoted by lowered apoplastic pH and elevated ROS gradients as well as the creation of a tip-focused [Ca^2+^_cyt_] gradient stimulating actin reorganization and polar exocytosis of wall polymers to the tip (Zhang et al. 2022a, 2022b). One of FER's roles involves its association with LLG1 in root hair tips, activation of a specific associated ROP that further activates NADPH oxidase leading to apoplastic ROS production (Duan et al. 2010; Li et al. 2015; Zhang et al. 2022a, 2022b) (Fig. 15C; right). Another CrRKL1 root hair receptor called ERULUS is auxin induced, positively controls the phosphorylation of FER, and affects pectin dynamics through activation of PME activities (Schoenaers et al. 2018). Ground-breaking work that followed (Schoenaers et al. 2023) showed that RALF22 is expressed specifically in root hairs and primarily located in rings in the cell wall attached to a LRX1 (Fig. 15C, right). These studies also indicate that RALF 22 is not only a signaling peptide but also functions as part of a structural component that regulates the state of HG condensation. From their results, the authors envision an oscillation in which ME-HG, RALF22, and LRX1 are secreted at the tip during the low-pH wall-loosening phase, then RALF22 interacts with FER-LLG1 to inhibit the H + ATPase, and wall pH rises leading to the growth inhibition phase with increased PME activity that leads to a rise in DE-HG. DE-HG then binds to a separate complex of RALF22 with LRX1, leading to wall stiffening and formation of the ring structure that fixes the direction of expansion. The authors hypothesize that the wall stiffening may be a mechanosensory signal to the FER-LLG1-RALF22 complex as part of the growth cycle. More recently, a process strikingly analogous to the novel work described by Schoenaers et al. (2023) for Arabidopsis root hairs was reported for pollen tubes by Moussu et al. (2023; see also Mohnen 2023). In pollen tubes, 2 molecules of RALF4 were shown to interact with 2 LRX8 molecules to form a positively charged heterotetramer at the pollen tube tip that can then interact with DE-HG to form organized structural complexes in the shank wall that play a very similar role to that in root hairs determining wall strength and shape.

Only in retrospect, through collectively re-examining the examples chosen for Fig. 15, did it occur to us that the pleiotropic character of FER must relate to its key role in balancing the strength of the outcomes of signaling pathways for growth (Fig. 15, B and C), hypo-osmotic (tensile) versus hyper-osmotic stress (Fig. 15D), and defense (Fig. 15, A and E). In a recent thought-provoking paper, Malivert and Hamant (2023) discuss the many different phenotypes generated by *fer* mutants to try to identify a unifying feature that could underlie FER functions. They discuss many interesting options but seem to favor the idea that FER is fundamentally a mechanosensor modulated by different co-receptors and ligands such as pectins/OGAs and RALF peptides leading to changes in pH, Ca^2+^ and/or ROS that function to modulate the mechanical integrity of the cell.

##### Other receptors involved in CWI signaling

In a study that has defined an important experimental system for interrogating cell wall signaling, Herman Hofte and colleagues recognized that a number of different mutants of Arabidopsis impaired in cellulose synthesis either through mutations in CesAs or associated proteins such as KORRIGAN or by use of the cellulose synthase inhibitor ISX all showed a set of characteristic responses that include production of ectopic lignin, alterations in levels and structure of pectins, reduction in cell elongation, as well as increases in JA production. In a search for suppressor mutations of *Procuste* (*cesA6*), they identified a new, widely expressed CrRLKL1 that they termed THESEUS1 (THE1), “a character from Greek mythology who slew the brigand Procustes” (Hematy et al. 2007) (Fig. 15D). Importantly, they found that loss of THE1 sensing could attenuate the growth effects and production of ectopic lignin caused by the *cesA6* mutation and some other, but not all, *cesA* mutations (in 2007, it was not known that ISX does not inhibit the CSLD CSCs, and this needs to be kept in mind when reading papers that assume ISX is completely inhibiting all the cellulose synthesis). Gonneau et al. (2018) have shown that binding of RALF34 to THE1 promotes cytosolic [Ca^2+^_cyt_] transients and cell wall alkalinization similar to that observed for RALF1 and FER (Fig. 15D). The *fer 1-4* or the *the1/fer 1-4 double* mutant lacked such responses to both RALF1 and RALF34 as well as the same effects of both peptides on upregulation of specific genes, suggesting THE1 may act upstream of FER or in parallel in a FER-dependent way.

Mechano-sensing is increasingly recognized as playing a key and perhaps essential role in CWI sensing (Hamant et al. 2019; Bacete and Hamann 2020). Loss of cellulose synthesis can create tensile stress that can affect mechano-sensitive Ca^2+^ channels, and THE1 acts upstream of the MCA1 ion channel that senses hypo-osmotic stress (Engelsdorf et al. 2018), while FER, through binding of OGAs to its malectin-like domains, promotes activity of a different Ca^2+^ channel that opens in response to hyper-osmotic conditions (Feng et al. 2018) (Fig. 15D). Such stress can also lead to microtubule disorganization that is controlled by FER-dependent and -independent pathways (Hamant et al. 2019; Malivert et al. 2021) (Fig. 15D). An exciting new study has tied together the functioning of THE1 with the interactions between sensing of CWI affected by swelling and PM stretching akin to hypo-osmotic stress, as opposed to drought or salinity that cause hyper-osmotic stress that causes membrane contraction and plasmolysis. Bacete et al. (2022) showed that in ISX-treated cells, THE1 modulates both cell wall stiffness as measured by Brillouin microscopy and synthesis of JA, associated with CWI sensing. In contrast, treatment with sorbitol (osmotic stress) stimulated not JA but ABA production, while, with both ISX and sorbitol, JA synthesis occurred but ABA synthesis was inhibited. Stimulation of JA synthesis was under control of a protein called RLP12 that was only expressed in the presence of both sorbitol and ISX (Fig. 15D). Also important was that sorbitol-induced ABA production was not observed in protoplasts, indicating that it involves PM wall connections—most likely the Hecht's threads observed in plasmolyzed cells (Yoneda et al. 2020). Overall, it appears that THE1 is a positive regulator of JA synthesis and negative regulator of ABA synthesis (Fig. 15D). THE1 can also work in concert with another receptor kinase, HERCULES, and FER to mitigate the stress of heavy metals on cell elongation (Richter et al. 2017) and with the LRR kinase FEI2 to induce JA synthesis associated with CWI. Because THE1 is not required for pattern-triggered immunity, it is likely specifically involved in CWI signaling (Engelsdorf et al. 2018). However, Pogorelko et al. (2013) and Bacete et al. (2018) have made clear in their reviews that there are many examples of significant cross-talk between CWI and defense signaling that are not reviewed here.

A direct role of oligosaccharins in defense signaling first became apparent from the important early discovery that they are sensed through their interaction with wall-associated receptor kinases called WAKs (Kohorn et al. 2006, 2021b), leading to induced defenses through activation of downstream MAPK1 signaling pathways (Fig. 15E). In addition to WAKs, many other receptors (including FER; see Fig. 15A) are involved in immune signaling (Narváez-Barragán et al. 2022). The pathogen-related expression pattern of a cluster of 5 other *CrRLK1L* genes suggests a specific role for other CrRLK1Ls in the interaction with pathogens (Lindner et al. 2012). Another interesting example involves the Arabidopsis immune peptides called Peps that are widely distributed in the plant kingdom (Lori et al. 2015); the PROPEP genes are induced by signals such as OGAs, Peps, and defense hormones. Peps bind to a receptor complex of PEPR and other co-receptors, setting off a series of classic immune responses and/or repressing CWI signaling (Shen et al. 2019; Dressano et al. 2020).

#### Signaling involving SCWs

Although mostly studied in the context of PCWs, signaling may also be triggered following modification of SCWs. For example, reduced growth associated with ectopic activation of defense responses, particularly synthesis of pathogenesis-related (PR) proteins, has sometimes confounded attempts to reduce recalcitrance of SCWs to digestion through reduction of lignin content or modification of lignin composition (Gallego-Giraldo et al. 2011, 2018). Defense trade-offs have been suggested as 1 possible explanation. This is not always the case, because some suppressor mutants of the growth reduction in plants with reduced lignin levels still exhibit defense gene expression (reviewed in Ha et al. 2021). The recent demonstration that some PR proteins are themselves cell wall localized, and function via generating small peptide defense signals (Chen et al. 2020a, 2020b), suggests that PR protein induction could be viewed as part of a broader program of cell wall remodeling that occurs following genetic modification of cell wall polymers.

Although pectin is considered a quantitatively minor component in SCWs and xylan the main wall component interacting with lignin (Kang et al. 2019), emerging evidence points to the possibility that pectin is a critical part of an underlying SCW structural scaffold that encompasses multiple wall components, including xylan and lignin (Hao and Mohnen 2014; Li et al. 2019). Modification of pectin through downregulation of GAUT4 increases both growth of and saccharification efficiency in switchgrass, the latter being a characteristic result of lignin reduction in SCWs (Biswal et al. 2018). Conversely, reduced lignification (e.g. in the *cinnamoyl CoA reductase 1* mutant of Arabidopsis) causes solubilization of pectic elicitors that activate production of a suite of *PR* genes (Gallego-Giraldo et al. 2020). This process involves the FERONIA-dependent transcriptional activation of ADPG1 (Liu et al. 2023), a polygalacturonase normally associated with anther dehiscence, a process that is also dependent on correct lignin deposition (Dai et al. 2019). The ectopic expression of ADPG1 is not itself responsible for the initial release of pectic material from the wall but rather with its processing to elicitor-active moieties, which are then recognized by WAK receptors leading to induced defenses (Gallego-Giraldo et al. 2020; Liu et al. 2023) (Fig. 15E).

#### Coordinating signal pathways for growth, development, and stress

Clearly activities that occur within the cell wall sit at 1 nexus that controls the regulation of growth, development, and responses to stress. All the processes of cell wall biosynthesis and secretion and polymer modifications and interactions, when functioning normally, are essential to carry out the complex programs involved in plant growth and development. This section has addressed the ways in which cell walls sense when normal growth is disrupted and set in motion processes designed to mitigate such stresses and that these processes rely on similar or sometimes identical ligands, receptors, co-receptors, and downstream processes used for growth. The wall as a shapeshifter is made even more clear in the final section that will examine the changing nature of the cell wall as it plays a key role in coordinated patterns of cell division, expansion, maturation, and programmed cell death (PCD). Again, we shall see that hormones play key roles as input and output signals, and the levels of both intracellular and extracellular Ca^2+^, ROS, and pH are recurring key actors. Understanding how the signals involved in all these events are coordinated is certainly one of the future challenges facing cell wall signaling.

### The life of a cell wall

Although not often discussed, cell walls have 4 distinct life stages: birth at the cell plate, tip, or diffusive growth, SCW maturation, and sometimes prolonged death long after that of their parent cells. Cell plate, tip growth, and maturation show some interesting and rather surprising similarities in terms of wall deposition. The outlier—the process of expansion growth—is one of most-studied processes in all of plant biology and has always seemed different and perhaps the most complex. This section examines these stages, placing emphasis on similarities and differences in wall structure and function between the life stages.

#### Birth at the cell plate

Cell walls are born during the process of cell division which involves building a new wall where none existed before. Obvious places of birth are stem and root apical meristems, but there are other important meristems such as those in leaf primordia and the cambium. Work by Peter Hepler on the dynamic behavior of the cytoskeleton recognized the importance of temporal and spatial changes in intracellular Ca^2+^ to cell plate formation and tip growth (Hepler 2005). Andrew Staehelin, who coupled a strong intellect to interpretation of his outstanding EM images, contributed greatly to our understanding of membrane dynamics and secretory processes. Hepler and Staehelin's work and several seminal reviews laid the foundation for our current understanding of cell plate formation (Samuels et al. 1995; Staehelin and Hepler 1996).

The initial establishment of a Ca^2+^ gradient focused on the developing cell plate is essential to the process, and conditions that dissipate this gradient disrupt cell plate formation (Staehelin and Hepler 1996). Ca^2+^ is important for cytoskeletal dynamics, vesicle fusion, callose synthesis, and pectin structure, all relevant to cell birth. During anaphase the spindle MT array gives way to a new MT array that defines the central position of the cell plate; actin microfilaments also form in this region and both are oriented with their plus ends to the center. This growing structure, called the phragmoplast, becomes a complex array of MTs, actin microfilaments, ER- and Golgi-derived vesicles (Samuels et al. 1995), and/or PM-derived endocytotic vesicles (Baluska et al. 2003) that delivers materials necessary for wall formation and is dismantled upon completion of the new wall. Vesicles fuse to form tubular structures that become the new PMs separated by the newly deposited wall.

**Figure 16.**
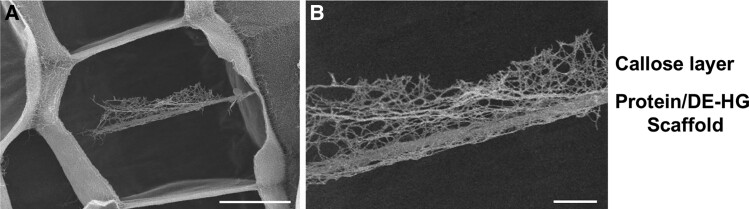
The developing cell plate in Arabidopsis. **A)** The direction of the Ca^2+^ gradient. **B)** An expanded version of **A)**. Modified from Fujita and Wasteneys (2014) to indicate the calcium gradient and the positioning of a proposed location of a callose layer and a scaffold.

Extensin3 (EXT3) was proposed to be deposited and cross-linked to form a scaffold on which to build the cell plate (Cannon et al. 2008). This has recently been questioned (Doll et al. 2022), although it is clear that, in the presence of extensin peroxidase, EXT3 undergoes self-recognition and polymerization into rope-like and cross-linked dendritic assemblies via end-on and lateral association, as revealed by AFM (Fig. 10D). ME-HG is also deposited early and is gradually demethylated, but whether DE-HG interacts with the positively charged EXT3 or some other similarly charged protein to form a “scaffold” requires further examination. Moore and Staehelin (1988) detected xyloglucan showing abundant labeling early in vesicles surrounding the developing plate and also within the plate. Callose is then detected in the well-developed tubular structures in a rare case where it serves as a wall component instead of defense polymer. Callose is by far the most abundant polymer of cell plates, and studies with an inhibitor of synthesis indicate that it is absolutely essential (Park et al. 2014; Drakakaki 2015). Figure 16 shows an impressive image of a developing plate from Arabidopsis that we suggest appears to show 2 different layers that might represent the early scaffold followed by a callose-rich layer.

Cellulose deposition begins sometime after callose synthesis, and the process differs from that in elongating cells because it involves CSLD proteins. In Arabidopsis cell plates, Gu et al. (2016) reported early deposition of cellulose by AtCSLD5 while AtCESA3 was more active at a much later stage, and once the new wall merges with the parent wall, CesA proteins can be seen migrating to the new wall; other reports indicate some overlap in CSLD and CESA expression (Miart et al. 2014; Chen et al. 2018). Wu et al. (2023) have shown that, in moss protonema that display both tip growth and cell divisions, PpCSLD6 (and not CesA) is active in both tips and cell plates; it was also sensitive to DCB but not ISX providing an easy way to distinguish CSLD from CESA activity.

At present, it is not clear why CSLDs are the preferred cellulose synthases for both cell plate and tip growth. One possibility is that CSLDs, but not CESAs, are tolerant of the high Ca^2+^ gradient at the center of cell plates and also at the tip of root hairs and pollen tubes. It may also be important that the products of CSLD synthesis are less oriented and/or less crystalline (Yang et al. 2020b). There can be interactions between β-1,3-glucans and cellulose that result in changed properties in hydrogels or aqueous solutions (Abou-Saleh et al. 2018). Such a hybrid network may be more easily produced when the cellulose MFs are more disordered and less crystalline and such a new composite may be more suitable for the new wall—an area rich for further investigation. As the cell plate matures and joins the parent wall, it is reported that callose is digested (Drakakaki 2015), although it is not entirely clear whether some or all may be masked by the beginning of “normal” wall synthesis.

#### Growth

##### Tip-growing cells

The major targets for study, the pollen tube of Lily and both the pollen tube and root hair of Arabidopsis, have been excellently reviewed (Cheung and Wu 2011; Rounds and Bezanilla 2013; Johnson et al. 2019; Çetinbaş-Genç et al. 2022). This discussion focuses mostly on root hairs and some of the interesting similarities with cell plate formation. The deposition of wall polymers at the tip also occurs where no wall exists and relies upon the establishment of a [Ca^2+^_cyt_] tip-focused gradient but with rapid (∼30 s) extracellular oscillations that parallel those of protons and ROS but lag slightly behind the auxin-induced oscillations in growth (Monshausen et al. 2007; Zhang et al. 2022b). Wall construction begins with deposition of ME-HG at the tip that gradually becomes DE-HG through the action of PMEs. Beat Keller's group (Baumberger et al. 2001) discovered that the leucine-rich extensin LRX1 becomes insolubilized in root hair cell walls, providing a very early suggestion that it may function as a scaffold along with DE-HG. Xyloglucan is also deposited early, and it is interesting that an unusual acidic xyloglucan (that potentially could also interact with LRX1) is deposited in root hairs but not pollen tubes (Pena et al. 2012). As for cell plates, callose deposition begins later, but with some differences: root hairs are relatively richer in cellulose, although they do deposit some callose, while pollen tubes and cell plate walls are very rich in callose [although some is found in callose plugs in pollen tubes (Kapoor and Geitmann 2023)]. Pollen tubes were one of the first places where CSLDs were shown to function (Doblin et al. 2001) but, in the same year, Favery et al. (2001) showed that the KOJAK gene that encodes AtCSLD3 is necessary for root hair growth. Subsequently it was shown that AtCSLD2 and AtCSLD3 but not CESAs function in cellulose synthesis in root hairs of Arabidopsis (Bernal et al. 2008: Park et al. 2011).

Signaling networks for tip growth are complex and involve many of the players described for CWI signaling as well as those used for diffusive growth (Kroeger et al. 2008; Zhang et al. 2022b). As previously discussed (see Fig. 15C), Schoenaers et al. (2023) provide perhaps the best description yet of oscillations in signals and wall structure that promote either wall-loosening or stiffening and correlate with oscillations in growth in root hairs.

##### Expansion (diffusive) growth

Studies of expansion growth began with the discovery of auxin by Fritz Went and have continued on for decades by other pioneers such as James Lockhart, Bob Cleland, and Dan Cosgrove (see Du et al. 2020b for recent review). To reiterate briefly, the driving force for growth is turgor, and, if cellulose MFs are aligned perpendicular to the axis of growth, the cell elongates. Auxin promotes wall acidification through activation of PM H^+^-ATPase (AHA2) that leads to “acid growth” through wall-loosening that creates new “space” that is filled in by deposition of new wall polymers. Wall remodeling (stiffening) that follows is assumed to involve mechanisms that work in opposition to wall-loosening.

To take a new look at expansion growth, we ask whether it is fundamentally not so very different from tip growth, as has also been implied by others (Wolf and Hofte 2014; Wolf et al. 2014; Höfte 2015; Jobert et al. 2023). It seems not too difficult to imagine such growth as an oscillation between a loose wall and a stiff wall. In a surely oversimplified scenario for diffusive growth, the loose wall has a lower pH, is relatively enriched in newly synthesized polymers (and therefore should initially have a high ratio of ME-HG to DE-HG), has a low level of cross-linking of pectic and/or protein polymers, and newly deposited cellulose is kept from interacting with itself and other polymers by expansin. The stiff wall follows in rapid succession as pH rises, ME-DG is converted to DE-HG promoted by PME, much more stiffening occurs through Ca^2+^-bridges and/or HG condensation through interaction with positively-charged proteins, peroxidase-mediated activity, and borate cross-links for RGII. The concept of this “loose wall” and the “stiff wall” could easily be consistent with oscillations in Ca^2^, ROS (for crosslinking and redox control), new wall polymer deposition, and wall pH (activity of PME vs PMEI, expansin, and THE1 and FER are also all pH regulated). This concept is consistent with FER and THE1 being positive regulators of cell wall stiffness (Höfte 2015; Bacete et al. 2022). But we do not see these 2 different types of walls when we isolate growing tissues because we isolate a mixture of loose and stiff walls.

If this oversimplified scenario has any truth to it, then the larger questions are what feedback loops involving auxin and/or other signals regulate the oscillations, what is the period, where are they spatially located, and why do we not see the process as a growth oscillation? Perhaps because it is not as localized as is tip growth? Perhaps it might be regulated by oscillations of only seconds to minutes and localized to a multitude of growth sites? There is 1 apparent oscillation occurring in multiple places that we can actually see at a microscopic level in elongating hypocotyls: CESAs have a residence time and period of activity in the PM of 7 to 10 minutes after which they become inactive and are internalized where they are targeted for degradation or recycling. Later new and recycled CESAs are inserted along the same MT-aligned path. Assuming deposition of other wall polymers is also locally coordinated with cellulose deposition (Ganguly et al. 2020), this is a highly localized oscillation in wall deposition that would occur in a multitude of sites that would be difficult to detect. Hepler and colleagues argued that a measured oscillation in exocytosis of wall materials in tip-growing pollen was a driving force for oscillations in wall thickness and growth (McKenna et al. 2009). A cycle driven by an oscillation in auxin action leading to oscillations in wall pH, internal Ca^2+^ levels and ROS, might fit with an assumption that CESAs are inhibited by Ca^2+^ (something that needs direct investigation) and would require the substitution of CSLDs for CESAs when the Ca^2+^ gradient is tip focused or high at the cell plate and also promote callose synthesis. For such diffuse growth there may be no need for an LRX1-DE-HG scaffold complex or callose. LRX1 is specific for tip growth (Carol and Dolan 2002), and perhaps there is some mechanism to keep callose synthesis inhibited, 1 possibility being its association with the abundant Ca^2+^ -binding annexin (Andrawis et al. 1993).

Exciting recent work indicates feedback loops ranging from seconds to minutes between auxin, wall pH, Ca^2+^, and pectin remodeling, reviewed by Jobert et al. (2023). They suggest a possibility of special regulation within microdomains wherein there are feedback loops between the state of auxin transporters and cell wall structures. It takes a few minutes for auxin's effects on transcription to show up [e.g. as upregulation of key regulators such as ERU, H^+^ ATPases, and small auxin up-regulated RNAs (Du et al. 2020b; Jobert et al. 2023)]. But auxin can also cause very rapid (seconds) fluxes in wall pH in roots that are in opposition to the canonical regulation of AHA2 by auxin carried out through transcriptional regulation (Li et al. 2021).

If we could stop the process at one point in the cycle, would we then see a wall structure that is largely loose or stiff? Perhaps DCB-adapted cells with their pectin-based walls (Shedletzky et al. 1990, 1992) or the alternating pectin/cellulose wall layers in celery collenchyma cells (see Fig. 9) offer clues. Or perhaps walls with disturbances on a larger spatial scale such as during hypocotyl hook formation or gravitropism (Jobert et al. 2023). Stay tuned; acid growth has never seemed so exciting!

#### Maturation

Cell wall maturation is defined here as the production of a SCW that is traditionally said to consist of S-1, S-2, and S-3 layers. The process is assumed to begin when elongation of a cell ceases, but there is sometimes an interesting overlap between these 2 processes during deposition of the S-1 layer. This transition cell wall (TCW) is the most interesting, and the one to which we give most attention. In view of space limitations, we leave discussion of many other aspects of SCW development, including regulation of lignification, to others (Turner et al. 2007; Hobson et al. 2010; Li et al. 2016c; Meents et al. 2018; Rao and Dixon 2018; Liu et al. 2021). In some ways, the making of a TCW is akin to deposition of a new wall because it is different from the PCW and is laid down without integration of wall material into the PCW. Thus, maturation might use the TCW as a scaffold upon which to deposit new material.

The cotton fiber is a unique cell type and has many advantages for study of the development of cell walls (Haigler et al. 2012). It originates from epidermal cells of the ovule. However, evidence indicates that it displays both tip growth and diffusive growth along the length of the fiber (Qin and Zhu 2011). Fiber cells within a boll develop synchronously from the day of anthesis; fibers elongate (up to about 2 cm) and deposit a typical dicot PCW comprised mostly of cellulose, xyloglucan and pectins (Meinert and Delmer 1977). At about 16 days post-anthesis, TCW synthesis begins. The fiber continues to elongate while beginning the deposition of this new TCW called the “winding layer” that is analogous to the S-1 layer of wood cells. From about 24 days onward, the fiber deposits almost exclusively cellulose in S-2 and S-3 layers resulting in a wall that is >90% cellulose by weight at maturity.

The idea that the TCW serves as a scaffold occurred to us because we realized that there are some interesting similarities to wall deposition in cell plates and tip-growing cells. The beginning of the TCW is characterized by a remodeling of pectin resulting in a net loss of uronic acids from walls that occurs at the same time the middle lamella between fibers degrades (Meinert and Delmer 1977; Singh et al. 2009). The expression of 2 Rac/ROP GTPases occurs precisely during this TCW phase of fiber growth (Delmer et al. 1995). Potikha et al. (1999) demonstrated H_2_0_2_ production associated with the TCW stage, and early addition of H_2_0_2_ caused premature initiation of TCW formation while an inhibitor of NADPH oxidase inhibited initiation. Strikingly, these observations are very similar to those observed for initiation of tracheary element differentiation in Zinnia cell cultures (Karlsson et al. 2005). Roberts and Haigler (1989) also presented evidence for a rise in Ca^2+^ levels in tracheary elements during the transition, and significant callose deposition is initiated early in the TCW stage in cotton fibers concomitant with first signs of SCW cellulose, a key component of the winding layer and showing no evidence of turnover (Maltby et al. 1979). As far as we can tell, there is no report of callose in S1-layers of wood, although it is found in tracheids of compression wood where it is called Larcinan (Boyd 1978) and in the G-layer of flax phloem fibers (Ibragimova et al. 2017). Primary wall CesAs apparently continue to be active with a gradual transition to secondary wall CesAs during the TCW transition (Tuttle et al. 2015; Watanabe et al. 2018). CslDs are expressed at all stages of fiber development and their role, if any, needs clarification (Li et al. 2017). In sum, it appears that the cotton fiber winding layer (as a scaffold?) is initiated by elevation of levels of intracellular Ca^2+^/ROS, production of DE-HG followed by a callose-rich layer mixed with cellulose perhaps made by both PCW and SCW CesAs. There is other very interesting evidence that there may be a pectin-rich scaffold in cells of wood since transgenic modification of poplar to make less HG leads to acceleration of growth and improvement in digestibility of the walls (Biswal et al. 2018). This may also relate to the finding that overexpression of an enzyme that degrades RGI enhances degradation of the middle lamella and also enhances growth in poplar (Yang et al. 2020a).

As for the later synthesis of the S-2 and S-3 layers, synthesis of xylans, glucomannans and lignin have already been discussed. CSCs increase significantly in numbers and velocity of movement and become more aggregated (Watanabe et al. 2015). Cotton fibers, bast fibers, and tracheary elements all share the same primary transcriptional regulation and the same set of genes is involved in SCW cellulose synthesis. An early transcriptome analysis to identify a common gene network for SCW synthesis in cells such as cotton fibers, xylem, and cellulose-rich tension wood identified 52 genes that met a very wide range of criteria (Ko et al. 2006). Among the genes on the list are NAC and myb TFs, genes encoding enzymes for all the major SCW polymers and, interestingly, also pectin synthesis. Not surprisingly, the cellulose-rich cotton and bast fibers all downregulate the transcription factors that induce deposition of lignin and hemicelluloses (Tuttle et al. 2015).

SCW synthesis represents a major carbon sink in maturing cells (Verbančič et al. 2018; Pottier et al. 2023). As discussed in those reviews, the source of the UDP-Glc that is the substrate for both cellulose and callose synthesis still remains controversial for some. For cotton fibers that make only callose and cellulose and little or no starch during SCW synthesis, the carbon comes from sucrose that enters the fibers through plasmodesmata. Amor et al. (1995) showed that 80% of the highly expressed sucrose synthase (SuSy) becomes associated with the plasma membrane fraction during SCW callose and cellulose synthesis, and Salnikov et al. (2001) also showed similar PM localization in developing zinnia tracheary elements. Amor et al. (1995) and Haigler et al. (2001) proposed this SuSy may channel carbon directly to cellulose or callose, a pathway that saves 1 ATP worth of energy for each Glc residue polymerized compared with the alternate pathway involving invertase and UDP-Glc pyrophosphorylase, In addition, recycling of the UDP (that inhibits glucan synthases) back to PM-SuSy would prevent its localized accumulation. This work also showed that supplied UDP-Glc can support callose but not cellulose synthesis in permeabilized fibers while sucrose can supply carbon for synthesis of both, another argument for possible in vivo channeling of UDP-Glc directly to the active site of CSCs. This PM-SuSy is most likely the same isoform found to be up-regulated by Arpat et al. (2004), and Brill et al. (2011) also showed strong up-regulation of a special SuSy in PM and wall fractions of cotton fibers starting during the TCW stage. However, as discussed by Verbančič et al. (2018) and Pottier et al. (2023), this role for SuSuy has been questioned, primarily due to mutant analyses showing that SuSy clearly is not absolutely necessary in Arabidopsis (Wang et al. 2022a, 2022b), and mutant analysis of cytoplasmic invertases, that may be important for more reasons than supplying carbon for cellulose synthesis, are essential for normal seedling growth (Barratt et al. 2009). However, both SuSy and UDP-Glc pyrophosphorylase are found in high levels during both PCW and SCW phases and both catalyze reversible reactions, suggesting important roles for each and that one should also be available to substitute when the other is absent. Verbančič et al. (2018) and Pottier et al. (2023) do conclude that there is certainly strong evidence to support an important role for PM-localized SuSy in SCWs. Winter and Huber (2010) have shown that SuSy becomes associated with actin upon Ca-dependent phosphorylation and, its association with actin directs CSCs to appropriate sites on the PM during SCW (Lei et al. 2012)]. Whether such associations might relate to possible channeling of carbon directly through the gating loop of CSCs in vivo deserves further study.

One key step has been missing from the life of the secondary wall—what are the signals that turn on the transcription factors? Clearly hormones are involved (Haigler et al. 2012; Demura 2013; Meents et al. 2018). Just as we were finishing writing this review, a landmark paper appeared that opens up new avenues for this area of research. Cai et al. (2023) have shown that different variants of WAK10, in combination with a brassinosteroid receptor and modulated by binding of OGAs of different sizes and affinities, control the expression of CesA 4,7, and 8 genes, but not lignin, in rice. This process leads to either taller or shorter rice with less or more secondary wall cellulose. Furthermore, breeding of rice has gradually selected a whole set of WAK10 variants leading to shorter or longer rice. This may tie in with the appearance of OGAs at the TCW transition that can bind to and activate WAKs. All of this invokes shades of the Green Revolution and suggests new approaches for regulation of synthesis of cell walls in other crops and particularly those used for biofuels.

#### Death

Just as the cell signaling community seems to revere Greek gods, the PCD community has fun asking “Was it murder or suicide? What were the murder weapons? Where did they put the corpse?” The emerging answers seem to be: “It was suicide, and, as we have seen for life, the chief weapons are Ca^2+^ and ROS (and probably FER as well) and, as for the corpse, it gets eaten.” Intracellular Ca^2+^ and ROS levels certainly rise as an early event of cell death (Roberts and Haigler 1989), and it may be that the regulation of SCW synthesis and PCD are coordinated. A 40-kD serine protease appears at initiation of SCW formation and builds up over time until it appears to serve as the same signal to induce PCD (Groover and Jones 1999). As for the corpse, in some cases the entire cell including the cell wall is digested, but the walls of cells like cotton fibers, bast fibers, vessels, and fiber cells of xylem all survive—sometimes for millennia—in the cotton robes and wooden caskets found in the tombs of the pharaohs.

There are 2 kinds of PCD: one that is developmentally programmed such as that seen for tracheary elements, and the other that is involved in defense against pathogens (pPCD). pPCD can be of the type that is involved in the hypersensitive response (HR-pPCD) to biotrophic and hemi-biotrophic pathogens, whereas another form might be called nec-pPCD in which necrotrophic pathogens digest everything, including walls. Being able to understand the differences between these is very important for devising proper defense strategies, but that gets beyond the scope of this review. The details of plant PCD are all still emerging; the reviews of Huysmans et al. (2017) and Ustun et al. (2017) summarize the overall state of our knowledge.

In sum, it is quite remarkable how the same signaling pathways can be modified in one way or another during the life of the plant and how important the cell wall is for that life. But the life story of most cell walls ends well. Most walls survive for a very long time, carrying out their many functions serving not only their parent plants but the entire global ecosystem.

## Challenges for the next 100 years

- What structures do the different wall polymer types have at the time they are synthesized and deposited in walls of specific cell types?
- What are the interactions between and among polymer, and how do these interactions change, both temporally and spatially, for walls with different structures and functions?
- How are all these processes regulated and coordinated?

## Data Availability

This historical review contains no new data.

## Dive Curated Terms

The following phenotypic, genotypic, and functional terms are of significance to the work described in this paper:

GAUT4 Gramene: AT5G47780
GAUT4 Araport: AT5G47780

LRX1 Gramene: AT1G12040
LRX1 Araport: AT1G12040

GAUT1 Gramene: AT3G61130
GAUT1 Araport: AT3G61130

AdPG1 Gramene: AT3G57510
AdPG1 Araport: AT3G57510

AHA2 Gramene: AT4G30190
AHA2 Araport: AT4G30190

IRX14 Gramene: AT4G36890
IRX14 Araport: AT4G36890

IRX9 Gramene: AT2G37090
IRX9 Araport: AT2G37090

ARAD1 Gramene: AT2G35100
ARAD1 Araport: AT2G35100

ARAD2 Gramene: AT5G44930
ARAD2 Araport: AT5G44930

CESA6 Gramene: AT5G64740
CESA6 Araport: AT5G64740

CESA1 Gramene: AT4G32410
CESA1 Araport: AT4G32410

FEI2 Gramene: AT2G35620
FEI2 Araport: AT2G35620

MUR3 Gramene: AT2G20370
MUR3 Araport: AT2G20370

CESA Gramene: Cellulose synthase
CESA Araport: Cellulose synthase

DCB CHEBI: CHEBI:943

ISX CHEBI: CHEBI:63956

adg1 Gramene: AT5G48300
adg1 Araport: AT5G48300

AHA10 Gramene: AT1G17260
AHA10 Araport: AT1G17260

xylan CHEBI: CHEBI:37166
