# Correction to: The plant cell wall—dynamic, strong, and adaptable—is a natural shapeshifter

**DOI:** 10.1093/plcell/koae266

**Published:** 2024-10-22

This is a correction to: Deborah Delmer, Richard A Dixon, Kenneth Keegstra, Debra Mohnen, The plant cell wall—dynamic, strong, and adaptable—is a natural shapeshifter, *The Plant Cell*, Volume 36, Issue 5, May 2024, Pages 1257–1311, https://doi.org/10.1093/plcell/koad325

The authors failed to acknowledge Simões et al. (2020) for the photograph shown in Figure 1C. The corrected Fig. 1C legend should read as follows: **C)** Suspension-cultured cells from the C4 plant sugarcane (*Saccharum* hybrid spp; example shown taken from Simões et al., 2020) or C3 plants such as Zinnia elegans (reviewed by Demura, 2013) can be induced to differentiate into tracheary elements, making them good models for study of xylem biogenesis.

Additional reference supplied:

**Simões MS, Ferreira SS, Grandis A, Rencoret J, Persson S, Floh EIS, Ferraz A, del Río JC, Buckeridge MS, Cesarino I** (2020) Differentiation of Tracheary Elements in Sugarcane Suspension Cells Involves Changes in Secondary Wall Deposition and Extensive Transcriptional Reprogramming. Front. Plant Sci. 11:617020. doi: 10.3389/fpls.2020.617020

